# A framework and review of evidence of the importance of coral reefs for marine birds in tropical ecosystems

**DOI:** 10.1002/ece3.70165

**Published:** 2024-08-21

**Authors:** Graeme S. Cumming, Nicholas L. James, Chia Miin Chua, Victor Huertas

**Affiliations:** ^1^ ARC Centre of Excellence for Coral Reef Studies James Cook University Townsville Queensland Australia; ^2^ Marine Biology and Aquaculture, College of Science and Engineering James Cook University Townsville Queensland Australia; ^3^ Oceans Institute University of Western Australia Perth Western Australia Australia; ^4^ O2 Marine South Townsville Queensland Australia

**Keywords:** Aves, fish, food web, foraging, ocean, seabird, trophic

## Abstract

As global heating and other anthropogenic influences alter tropical marine environments, it is unclear how marine bird populations will be impacted and whether their current roles in tropical marine ecosystems will change. Although marine birds roost and breed on tropical islands in large numbers, the direct trophic interactions between these birds and their prey across the tropics are poorly documented. We present a first framework for evaluating the dependence on and contributions of marine birds to tropical coral reef ecosystems and use it to examine the evidence for different kinds of interaction, focusing primarily on avian diets. We found 34 publications between 1967 and 2023 that presented a total of 111 data sets with enough detail for quantitative dietary analysis of tropical marine birds. Only two bird species out of 37 (5.4%) had diets of >50% coral reef fishes and only one, the Pacific Reef Egret, appeared to depend almost entirely on reef‐based production. Marine birds are also prey for other marine organisms, but insufficient data are available for quantitative analysis. Evidence for indirect effects of birds in tropical marine environments is stronger than for direct dependence on coral reefs, particularly in relation to nutrient concentration and the fertilisation impacts of guano on corals. Dispersal of propagules (e.g. seeds, spores, invertebrate eggs) by bathing, drinking, resting or foraging birds is under‐studied and poorly documented. Although the degradation of coral reefs appears unlikely to have a significant direct impact on food availability for most marine bird populations, indirect effects involving marine birds may be disrupted by global environmental change.

## Graphical abstract

We reviewed the dependence of marine birds on coral reefs, with a focus on trophic relationships. We found evidence for only a few avian species, particularly the Eastern Reef Egret, depending directly on reefs. Birds nonetheless interact with reef and reef‐based food webs in a diversity of ways, many of which are poorly understood and under‐studied.

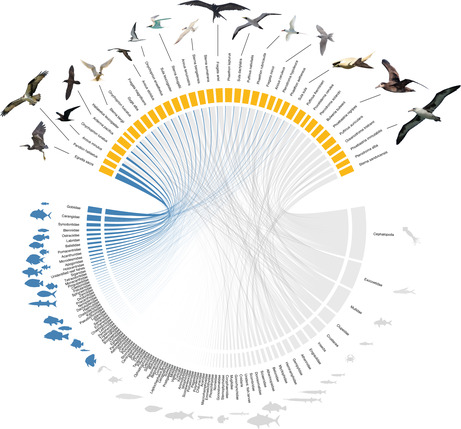

## 1 INTRODUCTION

Birds are conspicuous and abundant members of many tropical marine ecosystems, with large avian populations breeding and roosting on atolls and islands throughout the tropics. As tropical marine ecosystems and food webs globally experience a series of far‐reaching changes as a result of anthropogenic activities, tropical marine bird populations and the niches they occupy are increasingly coming under threat (Croxall et al., 2012; Dias et al., 2019). Globally widespread influences such as climate change, over‐fishing, introductions of invasive species, pollution and coastal development have profound implications for marine bird populations, their ecology, and conservation efforts.

Tropical marine birds (used here to describe both seabirds and birds that are strongly associated with the marine environment, such as Reef Egrets) face an uncertain future. Understanding how they will respond to global environmental change is made particularly challenging by the lack of established frameworks for describing and evaluating the different ways in which marine birds influence and are influenced by their biotic and abiotic environments. The ecology of birds in many primarily tropical marine and coastal habitats, such as coral reefs and mangroves, has received very little scientific attention despite the abundance of birds in these systems. For example, we do not currently have clear answers to such fundamental questions as how many bird species depend on coral reefs for food; the degree to which birds are prey for other marine animals; or which birds play important roles in dispersing the propagules of plants and other organisms between isolated coral reefs. Although some of these questions have been answered for individual species, no synthesis exists – suggesting a need for a review of this nature.

We evaluated the proposition that global changes in coral reef ecosystems will negatively affect tropical marine bird populations. Ongoing impacts of environmental change on coral reefs, and the vulnerability of corals to temperature rises in increasingly warm oceans, are already well documented (Ainsworth et al., 2016; Hughes et al., 2017; Siqueira et al., 2020). However, it remains unclear whether, and how, widespread reductions in coral reef integrity will impact marine bird populations and their biogeography. Most marine birds are known to forage offshore (refer to Table 1), but existing studies have generally not ruled out the possibility of additional, smaller‐scale foraging activities in near‐shore environments during periods of food stress. It is possible that dominantly offshore foragers may forage opportunistically on or near to coral reefs during periods of food stress, and that reef‐derived fishes might occasionally provide an additional source of nutrients for breeding marine birds in tropical waters. This possibility is supported by anecdotal observations, for example of Black Noddies appearing to forage between the beach and the reef crest on Heron Island (Cumming, pers. observ. 2020).

**TABLE 1:** List of publications presenting stomach content data from birds inhabiting islands and cays near coral reefs.

Authors	Title
Ainley et al. (2014)	The prey of Newell's Shearwater *Puffinus newelli* in Hawaiian waters
Alves et al. (2004)	Aves marinhas de Abrolhos – Bahia, Brasil
Ashmole and Ashmole (1967)	Comparative feeding ecology of sea birds of a tropical oceanic island
Asseid et al. (2006)	The food of three seabirds at Latham Island, Tanzania, with observations on foraging by Masked Boobies *Sula dactylatra*
Austin et al. (2019)	A sex‐influenced flexible foraging strategy in a tropical seabird, the Magnificent Frigatebird
Austin et al. (2021)	Interspecific and intraspecific foraging differentiation of neighbouring tropical seabirds
Bester et al. (2011)	Diet and foraging behaviour of the Providence Petrel *Pterodroma solandri*
Blaber et al. (1995)	Trawl discards in the diets of tropical seabirds of the northern Great Barrier Reef, Australia
Brown (1975)	Parental feeding of young Sooty Terns (*Sterna fuscata* (L.)) and brown noddies (*Anous stolidus* (L.)) in Hawaii
Catry et al. (2009)	Comparative foraging ecology of a tropical seabird community of the Seychelles, western Indian Ocean
Diamond (1974)	The Red‐footed Booby on Aldabra Atoll, Indian Ocean
Diamond (1975a)	Biology and behaviour of Frigatebirds *Fregata* spp. on Aldabra Atoll
Diamond (1975b)	The biology of Tropicbirds at Aldabra Atoll, Indian Ocean
Diamond (1983)	Feeding overlap in some tropical and temperate seabird communities
Donahue et al. (2021)	Genetic analysis of the diet of Red‐footed Boobies (*Sula sula*) provisioning chicks at Ulupa'u Crater, O'ahu
Harrison et al. (1983)	Hawaiian seabird feeding ecology
Harrison et al. (1984)	The diet of the Brown Booby *Sula leucogaster* and Masked Booby *Sula dactylatra* on Rose Atoll, Samoa
Jaquemet et al. (2008)	Comparative foraging ecology and ecological niche of a superabundant tropical seabird: the Sooty Tern *Sterna fuscata* in the southwest Indian Ocean
Le Corre et al. (2003)	Seasonal and inter‐annual variation in the feeding ecology of a tropical oceanic seabird, the Red‐tailed Tropicbird *Phaethon rubricauda*
Madden et al. (2022)	Foraging ecology of Red‐billed Tropicbird *Phaethon aethereus* in the Caribbean during early chick rearing revealed by GPS tracking
Madden et al. (2023)	Foraging ecology of Red‐billed Tropicbirds on Saba, Caribbean Netherlands, during early chick‐rearing
Monticelli et al. (2008)	Diet and foraging ecology of Roseate Terns and Lesser noddies breeding sympatrically on Aride Island, Seychelles
Nascimento and Azevedo‐Junior (2005)	Dietas das aves marinhas no Parque Nacional dos Abrolhos, Bahia, Brasil
Recher and Recher (1972)	The foraging behaviour of the Reef Heron
Schreiber and Hensley (1976)	The diets of *Sula dactylatra*, *Sula sula*, and *Fregata minor* on Christmas Island, Pacific Ocean
Seki and Harrison (1989)	Feeding ecology of two subtropical seabird species at French Frigate Shoals, Hawaii
Shealer (1998)	Differences in diet and chick provisioning between adult Roseate and Sandwich Terns in Puerto Rico
Smith (1985)	An analysis of prey remnants from osprey *Pandion haliaetus* and White‐bellied Sea‐eagle *Haliaetus leucogaster* feeding roosts
Smith (1990)	Factors influencing egg laying and feeding in Black‐Naped Terns *Sterna sumatrana*
Smith (1993)	Feeding and breeding of Crested Terns at a tropical locality – Comparison with sympatric black‐naped terns
Surman and Wooller (2003)	Comparative foraging ecology of five sympatric terns at a sub‐tropical island in the eastern Indian Ocean
Tayefeh et al. (2014)	Dietary segregation between breeding tern species on the Persian Gulf Islands, Iran
Villard et al. (2015)	Segregation in diet between Black Noddy (*Anous minutus*) and Brown Noddy (*A. stolidus*) from the southern lagoon of New Caledonia
Weimerskirch et al. (2004)	Foraging strategy of a top predator in tropical waters: Great Frigatebirds in the Mozambique Channel

To address this knowledge gap, we first propose a new framework that summarises how marine birds might be expected to interact with coral reef ecosystems. We then use our framework to interrogate the published scientific literature to identify (1) what evidence exists that each of the possible connections between birds and coral reefs might be important and (2) where further research is needed.

## 2 CONCEPTUAL FRAMEWORK

The potential interactions of coral reef ecosystems and marine birds are two‐way, with marine birds affecting the reef community and vice‐versa. Marine birds globally are known to play many potentially important ecological roles. These include the regulation of prey populations and participation in trophic cascades (Baum & Worm, 2009; Oppel et al., 2015); scavenging and waste removal (Sherley et al., 2020); the dispersal of plant and animal propagules (Aoyama et al., 2012; Burger, 2005); transfer of marine‐derived nutrients to terrestrial environments (Anderson & Polis, 1999) and concentration of nutrients (and plastics) at roost sites and on the fringes of oceanic islands (Caut et al., 2012; Gagnon et al., 2013; Graham et al., 2018; Grant et al., 2022; Lorrain et al., 2017); and the provision of cultural and psychological benefits to people, which in turn influence people's willingness to support and undertake conservation actions and protect natural environments (Jones et al., 2015; Moller et al., 2009). From a conservation and management perspective, given that marine birds respond to change across a range of spatial and temporal scales and levels of biological organisation, marine birds also serve as useful and relatively easily monitored indicators of change in the marine environment (Parsons et al., 2008). To date, however, it remains unclear whether they might serve an useful indicator role for coral reefs.

**FIGURE 1:**
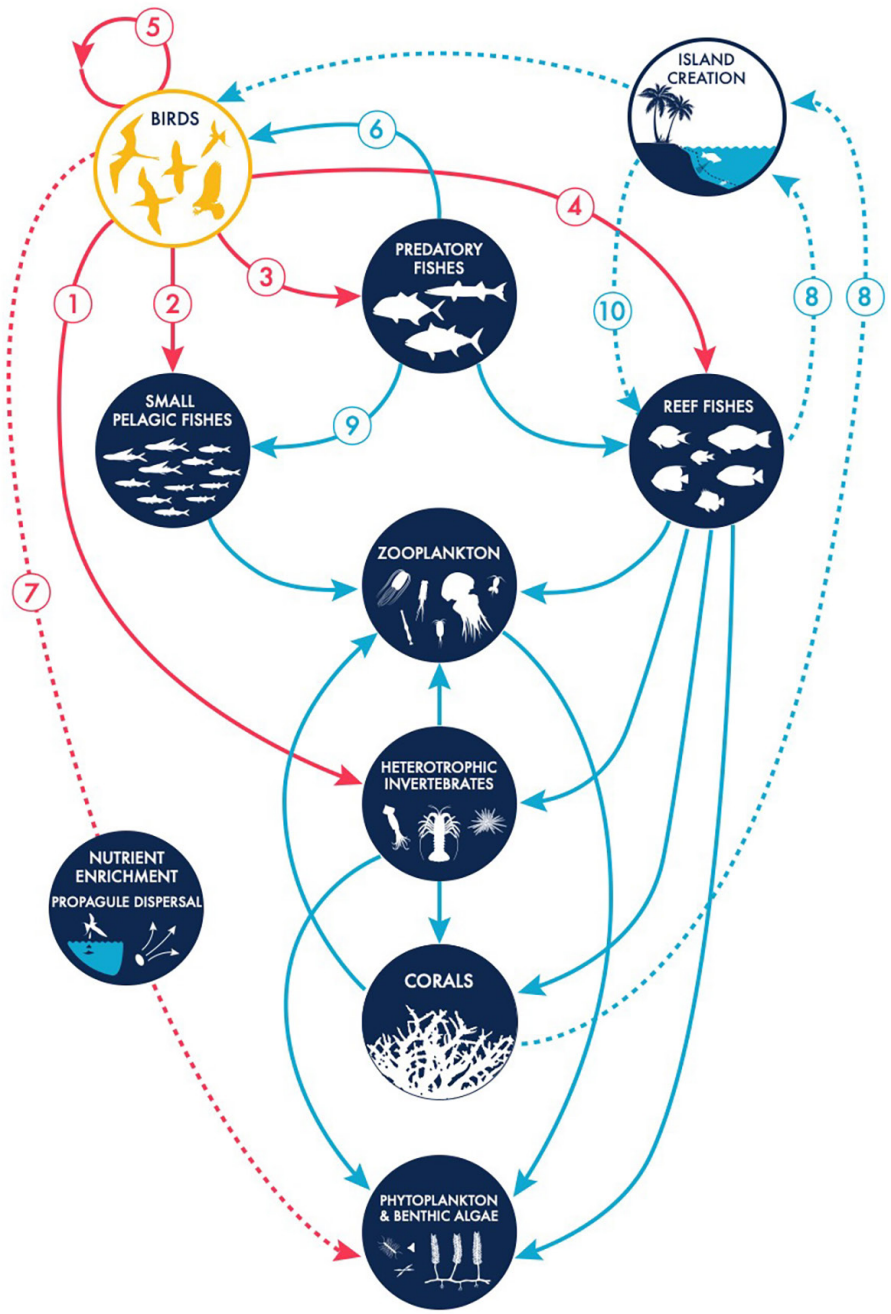
Conceptual framework illustrating different interactions in which birds may be involved within a tropical coral reef environment. Red arrows indicate direct (solid lines) and indirect (dashed lines) trophic effects of birds. Blue arrows indicate trophic effects originating in other taxa (solid lines) and indirect effects (dashed lines). The numbered arrows indicate (1) consumption of invertebrates by birds; (2) consumption of small pelagic fishes by birds; (3) consumption or competition between birds and predatory fishes; (4) consumption of reef fishes by birds; (5) consumption of birds by birds; (6) consumption of birds by predatory fishes; (7) indirect effects of birds on coral reef ecosystems, specifically nutrient enrichment and propagule dispersal; (8) indirect effects of corals and corallivorous fishes on birds, through the creation of atolls and islands that provide roosting and breeding habitats for birds; (9) consumption of small pelagic fishes by predatory fishes and (10) reefs' provision of structure to fishes.

Most recent conceptual models of coral reef ecosystems overlook birds, but a useful starting point is offered by a published biomass budget for major trophic pathways on coral reefs that includes interactions between birds, predatory fish (e.g. elasmobranchs, carangids and scombrids) and small pelagic fish (Polovina, 1984). Modification of Polovina's (1984) ecosystem model, with specific incorporation of coral reefs and bird‐derived nutrients, suggests a systems‐based overview that incorporates both direct and indirect effects (Figure 1, food web/flowchart).

We use Figure 1 as a framework to structure the remainder of this paper. Our goal was to evaluate existing evidence to determine which of these links can be considered significant in understanding the relationships between marine birds and coral reefs. Before describing our findings, we first explain how we approached the widely dispersed literature on this topic.

## 3 MATERIALS AND METHODS: LITERATURE REVIEW AND DATA CAPTURE

### 3.1 Literature review

We used a quantitative approach where sufficient evidence was available to do so and a more qualitative approach where evidence was lacking or widely dispersed. The main focus of the review was on understanding the dependence of marine birds on coral reef fishes as a source of food. This is the most direct and potentially most important interaction for understanding global change impacts on marine birds and one of the least researched. For this component, we conducted an exhaustive search for studies describing the stomach contents of marine birds inhabiting tropical shores in the vicinity of coral reefs.

**FIGURE 2:**
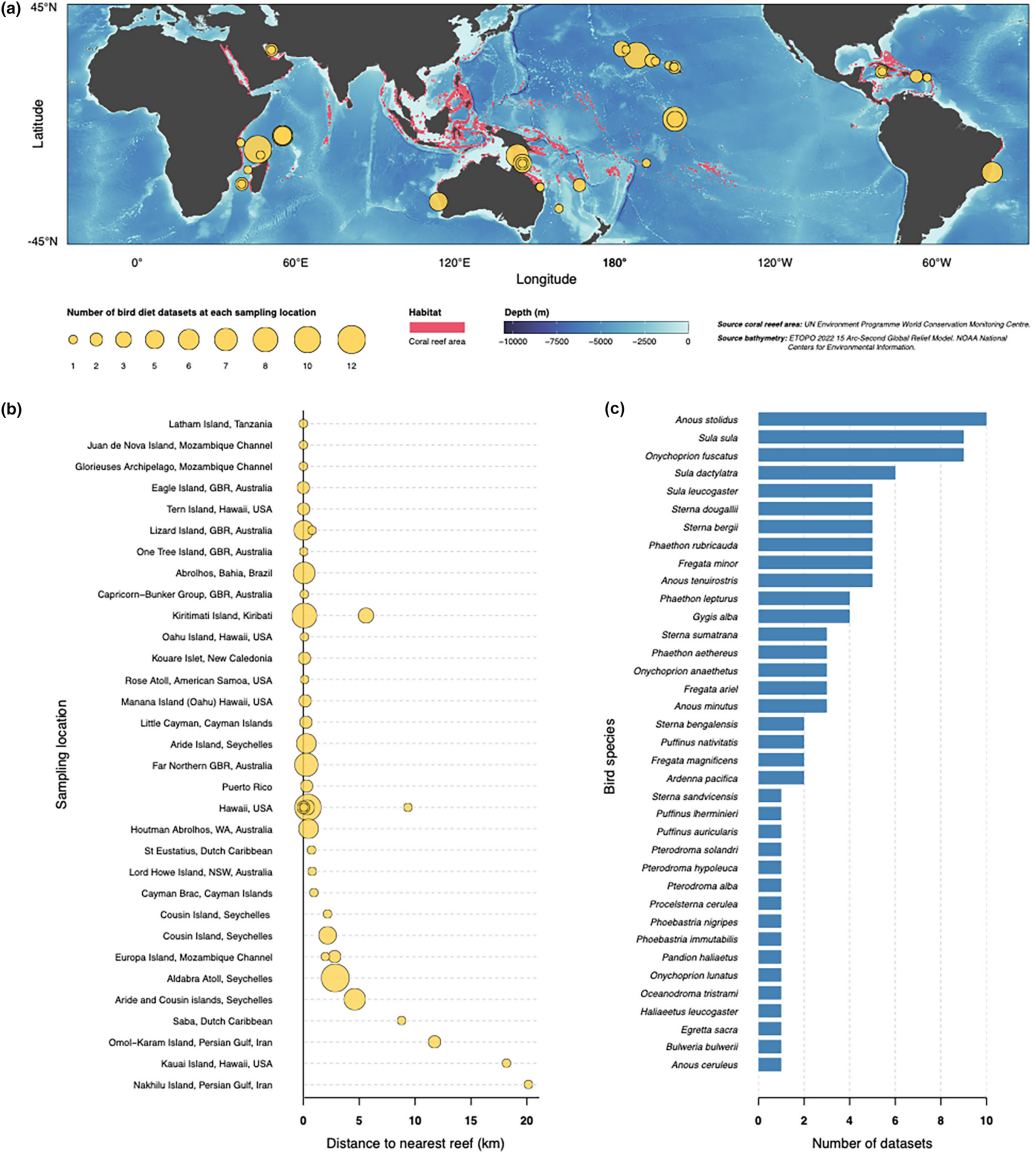
(a) Geographic distribution of sampling locations included in the meta‐analysis (1964–2022). Yellow bubbles indicate the geographic position of sampling locations and their size is proportional to the number of data sets at each location. Polygons in magenta represent the global distribution of coral reef habitat based on a data set compiled by the United Nations Environment Programme – World Conservation Monitoring Centre (UNEP‐WCMC et al., 2021). Bathymetry layer plotted from ETOPO 2022 15 Arc‐Second Global Relief Model. World map displayed using a Robinson projection. (b) Proximity of sampling locations to coral reef habitat. Each circle represents a site. Circle size is proportional to the number of data sets collected at each location. When sample collection for a bird species in a single study was spread across multiple islands, the primary location was represented. In these cases, the distance to the nearest coral reef is measured from the primary location. GBR: Great Barrier Reef; USA: United States of America; WA: Western Australia. (c) Number of published data sets for each bird species. A ‘data set’ comprises data collected for a single species at a specific location for the duration of a study (e.g. data on the diet of *Onychoprion fuscatus* at Aride Island, Seychelles, collected from 2005 to 2007 by Catry et al. (2009).

We first used the online database ISI Web of Science to search for studies published up to July 2023 that contained data on avian stomach contents (typically from regurgitated samples). Our specific search terms included a combination of the words ‘seabird*’, ‘diet*’ and additional terms with regions/nations where coral reef areas are present, such as ‘coral reef*’, ‘tropic*’, ‘Pacific Ocean’, ‘Indian Ocean’, ‘Caribbean’, ‘Bermuda’, ‘Red Sea’, ‘Great Barrier Reef’, ‘Coral Sea’, ‘Africa’, ‘Asia’, ‘Australia’, ‘Braz(s)il’, ‘China’, ‘Fiji’, ‘Florida’, ‘French Polynesia’, ‘Hawaii’, ‘Indonesia’, ‘Japan’, ‘Malaysia’, ‘Maldives’, ‘Micronesia’, ‘New Caledonia’, ‘Papua New Guinea’, ‘Philippines’, ‘Reunion’, ‘Seychelles’, ‘Samoa’, ‘Solomon Islands’, ‘Thailand’, or ‘Tonga’. Additional searches were undertaken to find studies that focused on Reef Egrets. We then ran the same searches in Google Scholar to identify additional suitable publications. Finally, we scanned the lists of references in all the publications found through online searches to identify additional publications with stomach content data. Dietary studies that only presented data obtained via stable isotope analysis or DNA metabarcoding were excluded because these techniques do not provide a precise number of identifiable prey items consumed; isotope analysis in particular lacks specificity in terms of prey species. In addition, there are relatively few such studies for tropical marine birds and the total number is insufficient for meta‐analysis. Overall, our exhaustive search for studies on the diets of tropical marine birds living near coral reefs identified a total of 34 studies published between 1967 and 2023 (Table 1). These 34 studies contained 111 data sets that had samples collected within 25 km of tropical coral reef habitat (Figure 2; full details and all data are in Appendix S1).

Although studies of movement ecology and at‐sea observations also offer valuable information about marine bird foraging locations and their potential dependence on reefs, an initial assessment of these studies suggested that they were too sparse and too un‐standardised in relation to the scale of assessment of foraging patterns to warrant a full formal review. Tropical marine birds are not widely studied in any case and of the telemetry studies that exist, a high proportion were not undertaken with a coral reef focus or at fine enough scales to determine whether any reef‐focused foraging occurred. We thus included ancillary information from tracking data in a brief written summary without further analysis.

Prey data from the literature were captured in a spreadsheet to the lowest taxonomic rank recorded in the original study. In most cases, fish remains in regurgitated samples were identified to family level or lower. All data were collected from colonies within 25 km of a coral reef (Figure 2b).

There were only five cases in which the fish remains could not be identified down to the family level; these were entered at the highest resolution that the authors were able to provide. These five prey categories were: ‘order Anguilliformes’ (eels), ‘order Beloniformes’ (needlefishes, barracudas and flyingfishes), ‘fish larvae’, ‘leptocephalus larvae’ and ‘unidentified reef fishes’. Leptocephalus larvae are pelagic larvae that can develop into fishes of several families of the superorder Elopomorpha, mainly eels (e.g. Anguillidae, Congridae and Muraenidae) but also tenpounders (Elopidae) and tarpons (Megalopidae). The larval stage typically occurs off the reef for all of these species (Miller, 2009).

We excluded fishes that could not be identified at all because it was not possible to determine whether they were reef‐associated or not. Although the data were predominantly comprised of fishes, we included additional prey groups such as cephalopods, crustaceans and other invertebrates that reflect the dietary breadth of marine birds. However, since these groups were not the focus of the study due to either their lack of coral reef association or their inaccessibility to birds on reefs, the data were pooled into higher taxonomic ranks such as Order (e.g. Cephalopoda), Class (e.g. Crustacea), or Phylum (e.g. Cnidaria). These data are presented in Appendix S1. While squids are an important dietary component for many marine birds, their occurrence in stomach contents is generally indicative of pelagic foraging on oceanic species rather than coral reef dependence. The majority of tropical squid and jellyfish (of a size that is suitable prey for birds) are pelagic species that are either not closely associated with coral reefs (Alabia et al., 2016; Arkhipkin et al., 2015) or occur on reefs at low densities. Many squid species favour open oceanic areas with higher currents (O'Dor et al., 2002). Reef‐associated squid (e.g. Bigfin Reef Squid *Sepioteuthis lessoniana*) are generally found below about 1.5 m, down to 100 m and move onto and off reefs during the night; larger aggregations of prey‐sized (i.e. non‐planktonic) squid are generally only present on tropical coral reefs during occasional spawning events (Chiang et al., 2020). Tropical diving birds such as gannets, darters and cormorants generally are not known to forage in coral reef habitats. Similarly, most Octopods on coral reefs would be unavailable to non‐wading birds and there are no planktivorous or gleaning birds associated with most coral reefs apart from Reef Egrets and a few opportunistic habitat generalists such as gulls, kites and oystercatchers.

We also undertook an exhaustive search for the second direct effect, ‘birds as prey’. Observations of fishes eating birds are usually published as small notes of interest within papers that have a different focus and so a very wide range of different search terms in combination with expert knowledge (e.g. ‘Hawai'i tiger shark bird’) was used to locate these articles. The nature of reporting for these data did not lend itself to a full quantitative or systematic analysis.

For the indirect elements of the framework, which are included for the sake of completeness but are not the primary focus of the manuscript, we have included representative references based on extensive but not exhaustive searches.

### 3.2 Data capture

We compiled data on the percentages of total number of prey ingested from published studies. These sources calculated ingested prey numbers from regurgitated samples based on counts of individuals that were, at times, complemented with counts of remains that identified a single individual (e.g. fish skull, fish caudal fin, squid beaks). This was the most prevalent metric found in the literature (76.5% of publications), allowing for the inclusion of a broader range of data sets. Most marine birds forage on quite specific size classes of fish, so the proportions of different fish species in their diet correlate strongly with the total number of each prey item. For studies where the percentage of prey items was not available but the number of prey items was recorded, we calculated percentages and included them in our data set. In some cases, data were presented for separate years or seasons. In such cases, we pooled the numbers of prey items together and calculated percentages by organism across all time steps. If the percentage of a given prey item was presented as ‘<0.1%’, this was included as ‘0.05%’ in our data set for use in quantitative analyses. One study used stomach content data to assess the presence of trawl discards in the diet of seabirds on the Great Barrier Reef (Blaber et al., 1995); in this case, we only included data collected away from trawling grounds or during the closed season. Another study in Australia (Blaber & Wassenberg, 1989) was excluded due to the proximity of the study area to known trawling grounds. Care was taken not to include data that had already been published in previous studies to avoid the presence of duplicates.

Once the data from suitable studies were selected, we validated and, when needed, updated the names of marine bird and fish species following the World Register of Marine Species (WoRMS; Ahyong et al., 2024), Avibase (Lepage et al., 2014) and FishBase (Froese & Pauly, 2023). The validation process was conducted using the functions ‘wm_records_names’ from the ‘worrms’ package (Chamberlain, 2020) and ‘validate_names’ from the rFishbase package (Boettiger et al., 2012) in R (see details in Appendices S2 and S3). In addition to the validation of species names, we also updated several names of genera and families to match FishBase records. For Blaber et al. (1995), we entered the genus *Mulloides* as its currently accepted name *Mulloidichthys* and *Paraexocoetus* as *Parexocoetus*. Three records from Ashmole and Ashmole (1967) were updated: *Caristium japonicus* was entered as *Caristius japonicus*, the family Mugiloididae was updated to currently accepted Pinguipedidae and the family Stomiatidae is now considered a subfamily within the family Stomiidae (thus family Stomiidae was entered instead). Three edits were made to records from Harrison et al. (1983): we updated the family of *Vinciguerria* sp. from ‘Gonostomatidae’ to ‘Phosichthyidae’ where this genus is currently placed; we updated the family name ‘Ostraciontidae’ to ‘Ostraciidae’ and we changed the family of *Ptereleotris heteroptera* from Gobiidae to Microdesmidae (although we note that there is an ongoing debate among taxonomists to resolve if microdesmids should be placed again as a subfamily within the family Gobiidae). Diamond (1983) provided data at a family level for Zanclidae. Since Zanclidae only contains one extant species (i.e. *Zanclus cornutus*), we added the genus *Zanclus* and species *Zanclus cornutus* to this record. Following name validation, prey items in the dataset were classified into three hierarchical taxonomic levels: family, genus and species. While some prey items were identified down to the species level and others were only identified to the genus level, the majority of prey items were identified to the family level. Thus, to ensure consistency and facilitate data analysis, all prey items were aggregated at the family level. This approach allows for a more comprehensive and standardised analysis, considering the varying levels of taxonomic identification.

In order to determine the reliance of birds on reef fishes for food, we classified the fish families found in their stomach contents as either reef‐associated or not, following the systematic approach adopted by Siqueira et al. (2020), who identified reef fish families as those where at least 20% of their members are listed in FishBase as ‘reef‐associated’. This criterion provides a broader list than a previous consensus list of reef fish families (Bellwood & Wainwright, 2002). We modified the resulting list by excluding mullids (goatfishes) because the mullids dominate the diet of multiple marine birds in the Seychelles area with >90% of their stomach contents (Catry et al., 2009; Diamond, 1983; Monticelli et al., 2008) are likely pelagic juveniles that occur off the reef (Catry et al., 2009). Therefore, although mullids are common reef‐dwellers (particularly as adults) and may have contributed to the diet of other marine birds in this study, we took a conservative approach and opted to exclude this group to avoid overestimating the degree of birds' reliance on reef‐derived fish production.

In total, the following families in our data set are listed as reef fishes: Acanthuridae, Antennariidae, Apogonidae, Balistidae, Blennidae, Caesionidae, Carangidae, Chaetodontidae, Cheilodactylidae, Cirrhitidae, Dactylopteridae, Diodontidae, Echeneidae, Ephippidae, Fistulariidae, Gobiidae, Haemulidae, Holocentridae, Kyphosidae, Labridae, Lethrinidae, Lutjanidae, Microdesmidae, Monacanthidae, Nemipteridae, Ostraciidae, Pempheridae, Pinguipedidae, Pomacentridae, Priacanthidae, Pseudochromidae, Scorpaenidae, Serranidae, Siganidae, Sparidae, Sphyraenidae, Syngnathidae, Synodontidae, Tetraodontidae, Tripterygiidae and Zanclidae. An additional category of ‘unidentified reef fishes’ recorded by Austin et al. (2019) was also included. When fish larvae had been recorded separately, we grouped the larvae with their respective family. Although the larval stages of the majority of reef fishes are pelagic (Leis & McCormick, 2002) and reef fish larvae were, therefore, most likely consumed off the reef, the removal of reef fish larvae from the system would both remove a source of food for birds and impact the reef fish community by reducing the number of new individuals settling onto the reef. To assess whether treating the larval stages of reef fishes as prey consumed off the reef would make a difference to our understanding of the dependence of birds on reefs, we also ran an analysis keeping the larval stages separate for comparison. The resulting plots are presented in the Appendix S3 (Figures S1 and S2). We assumed a Pomacanthidae (angelfish) record in Villard et al. (2015) to be of a pelagic larva, as the adults of even smaller species such as those of the genus *Centropyge* would probably be too large for noddies to feed on. Some studies may have grouped larval and post‐larval stages, but our analysis suggests that this would not have a significant impact on our conclusions.

We used only data sets that we deemed fully reliable and that met the criteria listed above for quality and detail. As explained below, we produced a series of simple descriptive plots from these data to explore trends in the foraging patterns of different tropical bird species on reef‐associated fauna. The data are provided in Appendix S1 and the R code used to run the analyses is in Appendix S2.

## 4 RESULTS AND DISCUSSION

Our findings are presented following the structure of the framework presented in Figure 1. We first consider direct trophic interactions: birds as predators and birds as prey. We then briefly consider indirect trophic effects, including cascading and behavioural effects for which little empirical evidence currently exists. Lastly, we consider the two primary ecosystem roles of marine birds: seed dispersal and nutrient concentration.

### 4.1 Direct trophic effects

#### 4.1.1 Birds as predators

**FIGURE 3:**
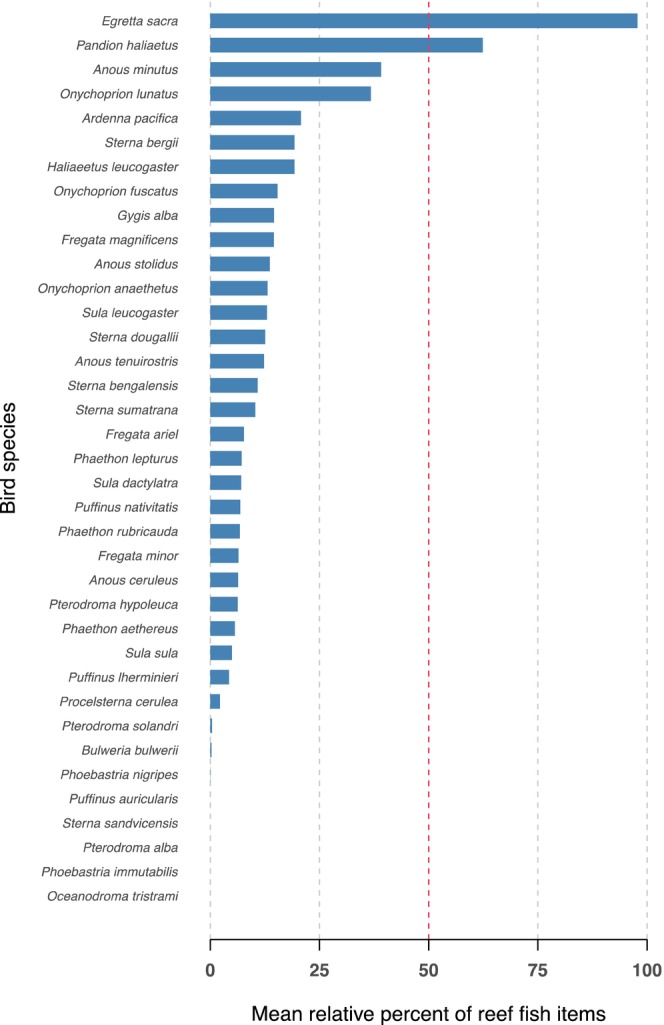
Mean relative percent of reef fish items in the regurgitated samples of coastal and oceanic birds that live in the vicinity of coral reef habitat (<25 km). Red line indicates that 50% of prey items consumed originated from a coral reef.

Focusing first on the direct interactions initiated by birds (indicated as red arrows in Figure 1), the most obvious potential dependence and impacts of birds on coral reef communities are as predators (Figure 1, arrows 1–4). A range of potential prey is available on reefs or in the immediate vicinity of reefs, including pelagic fish, juvenile predatory fish, reef fish, invertebrates and other birds. The collated data suggested that reef‐associated fishes are not the primary food source for most birds living in the immediate vicinity of coral reefs (Figure 3).

Only two bird species (Pacific Reef Egret *E. sacra* and the Osprey *P. haliaetus*) had more than 50% reef‐associated fishes in their diet during the period of study (Figure 3). Pacific Reef Egrets in particular showed the strongest connection of any bird species to coral reefs, with a large majority of their prey items being obtained directly from reef flats during periods when the tide is low (Recher & Recher, 1972). This result is consistent with published observations of Reef Egrets foraging on reef crests, in reef flat areas, and on the edges of rocky shores near to coral reefs (Recher, 1972; Rohwer, 1998). Reef Egrets living on coral cays are directly reliant on coral reefs; where more diverse habitats are available, we have also observed them foraging in mangroves, off rocky shorelines and human structures (e.g. sea walls, boats and moorings) and on mud flats (Cumming, pers. observ. 2019).

Small to moderate amounts of reef fish featured in the diets of another 25 bird species (Figure 3). Six additional bird species appeared to consume no coral reef‐derived fishes despite the proximity of their roosting and breeding sites to reefs. It is noteworthy that both Pacific Reef Egret and Osprey are relatively generalist foragers that are found in a wide range of habitats, including freshwater. The petrel and shearwater species that these data imply may be foraging near to coral reefs are more generally considered from movement studies to be open‐water pelagic feeders (McDuie et al., 2015; McDuie & Congdon, 2016; Weimerskirch et al., 2020).

Beyond direct dietary analysis, two additional strands of evidence about the dependence of birds on coral reefs are provided by movement tracking data and direct observations of foraging behaviour, respectively. Foraging bouts can be distinguished from commuting behaviour in movement data and experienced observers can usually tell whether birds encountered at sea are foraging based on their behaviours, particularly when species form foraging flocks. A full review of research findings in these areas is beyond the scope and objectives of this manuscript. Broadly speaking, however, few bird species have been observed foraging directly over coral reefs or within reef flat areas and tracking data suggest that the majority of birds breeding in atolls and tropical reef‐adjacent habitats are pelagic foragers. Tropical marine bird species observed breeding on tropical reef‐adjacent locations but not described in movement analyses as using reefs for foraging include at least 14 species: Red‐Footed Booby (Hays et al., 2020), Brown Booby (Correia et al., 2021), Masked Booby (Le Corre et al., 2012; Oppel et al., 2015), Great Frigatebird (Le Corre et al., 2012), Barau's Petrel (Le Corre et al., 2012; Pinet et al., 2011), Trindade Petrel (Trevail et al., 2023), Wedge‐tailed Shearwater (Le Corre et al., 2012; McDuie et al., 2018; McDuie & Congdon, 2016), Tropical Shearwater (Trevail et al., 2023), Red‐Tailed and White‐tailed Tropicbirds (Le Corre et al., 2012; Trevail et al., 2023), Sooty Tern, Brown Noddy and Lesser Noddy (Trevail et al., 2023) and Black Noddy (James & Cumming, 2024).

Austin et al. (2019) found that breeding Magnificent Frigatebirds exhibited flexible foraging strategies that included time spent in coastal zones and near fringing reefs around the Cayman Islands. They argued that a higher predictability of near‐shore resources might benefit breeding birds in particular, while also offering more opportunities for kleptoparasitism on other bird species breeding on land. Their analysis does not, however, specifically highlight reefs as being important foraging locations for frigatebirds.

**FIGURE 4:**
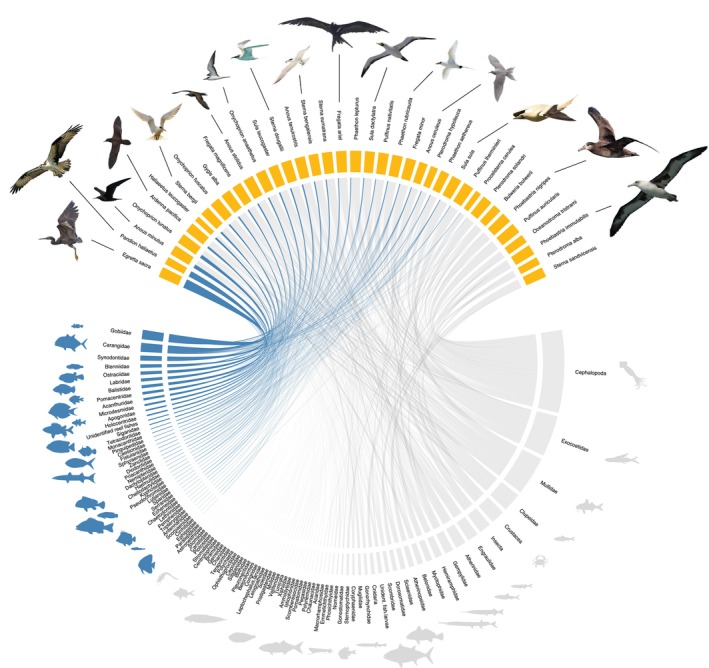
Mean relative importance (%) of reef‐derived prey items in the diet of 37 species of birds sampled on tropical islands within 25 km of coral reefs between 1964 and 2022. Yellow nodes represent birds, blue nodes represent families of reef‐associated fishes and grey nodes represent prey categories that are not reef‐associated. Link thickness is proportional to the mean relative percent of prey items found in the regurgitated samples of birds. Bird images credits: Alan Schmierer, Lawrence Milovich, Forest & Kim Starr, Michael Morel, Mike Prince, Eric Dale, JJ Harrison, Paul Harrison and Ariafrahman.

The bird species considered in our dietary data set include many of those that seem most likely to exhibit some form of reef dependence; thus, the addition of more tropical marine bird species would probably reduce rather than maintain the 5.4% proportion of reef‐dependent species that these data sets suggest. Although there is clear evidence that some birds regularly eat fishes that are widely considered to be reef‐associated, the direct connections between coral reefs as prey providers and birds as predators appear to be relatively weak. Looking in more detail at specific links between avian predators and fish prey (Figure 4) similarly suggests a relatively low reliance for most species on coral reef‐derived prey.

**FIGURE 5:**
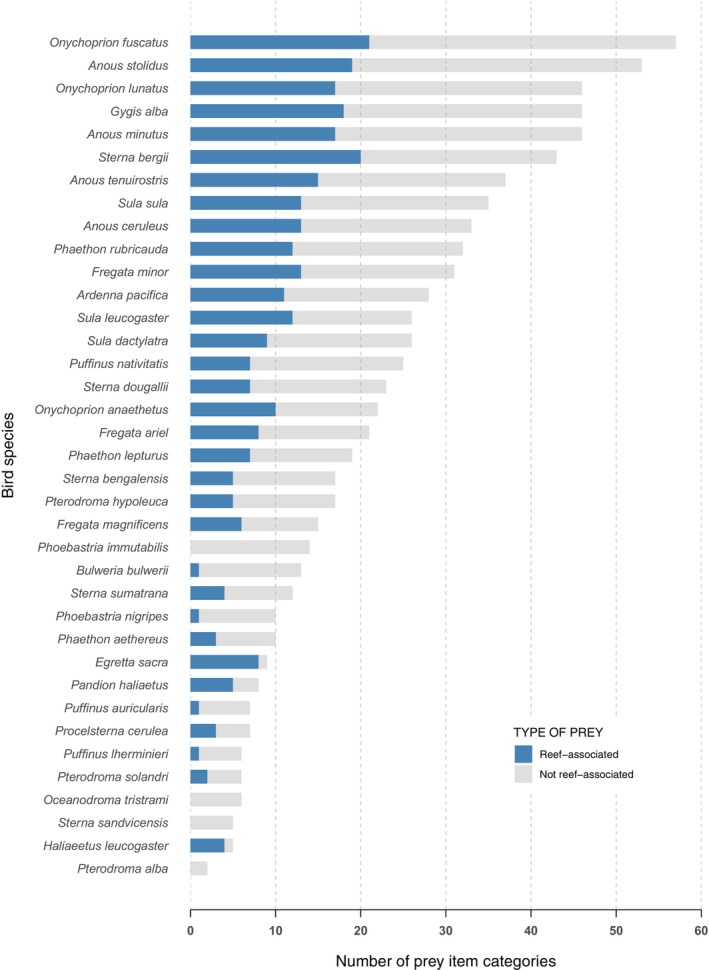
Dietary breadth of 37 coastal and oceanic birds near coral reef habitat. Prey categories indicate the number of distinct preys identified to the family level and, in some cases, higher taxonomic ranks (e.g. order), represented in the birds' diet. The larval and post‐larval stages of several fish families are presented together. Prey items are primarily comprised of fishes but they also include several groups of invertebrates. Reef‐associated prey are displayed in blue and prey that occur off the reef are in grey.

Bird species included in the study exhibited a range of different dietary breadths (Figure 5).

Most tropical marine birds show relatively low reliance on reef‐associated fish, suggesting that global declines in coral reef productivity are unlikely to have a direct effect on their prey base. Although the total proportion of coral reef‐derived prey items in marine bird diets appears to be generally low, these results do not rule out the potential for changes in dietary composition or preferences under different global change scenarios. It is possible that such changes may alter the relative importance of coral reefs for tropical marine birds. Specifically, coral reef fisheries may potentially be a source of resilience for breeding marine birds even if reefs only provide small amounts of accessible food during periods when pelagic prey are scarce.

#### 4.1.2 Birds as prey

Birds may also be prey for other birds and predatory fish, particularly sharks (Figure 1 arrows 5 and 6). Studies of shark impacts on birds have generally been from the perspective of shark researchers rather than bird researchers; records are scattered, but these observations indicate that predation occurs regularly enough to be considered more than a freak event. In some cases, the risk of shark predation may be high enough to influence the foraging behaviour of marine birds (Heithaus et al., 2009). Although most published observations have implicated tiger sharks, other shark species are also frequently associated with coral reefs and adjacent habitats (e.g. seagrass, macroalgae) and the scarcity of records of other shark species foraging on birds should not be interpreted as evidence for a lack of predation. Several studies have found concentrations of tiger sharks near seabird nurseries, where sharks feed on juvenile birds that are learning to fly. Analysis of a seasonal aggregation of Tiger Sharks (*Galeocerdo cuvier*) at French Frigate Shoals, northwestern Hawaiian Islands, suggested that sharks may eat up to 10% of all fledging Black‐footed Albatross (*Phoebastria nigripes*) from the colony (Meyer et al., 2010). In a study of seabird predation by Tiger Sharks in Western Australian waters, there was a 13.1% occurrence of seabirds in the stomach contents from 225 sharks, but the study did not identify the bird species (Simpfendorfer et al., 2001). Ingested seabird remains were found in 10.4% of 558 Tiger Sharks caught near Townsville between 1964 and 1986 (Simpfendorfer, 1992). A total of 2.6% occurrence of seabirds was found in the stomach content of 98 tiger sharks off Northern Australian waters, with albatross recorded as a prey item (Stevens & McLoughlin, 1991). A partial wing of an American Coot was discovered in the regurgitated stomach content of a tagged Tiger Shark off the Florida Keys (USA) (Gallagher et al., 2011).

Off Reunion Island, east of Madagascar, the analysis from the stomach contents of 30 Tiger Sharks found 4.73% contained *Gallus gallus* (Red Junglefowl) and 2.03% *Anous* sp. (noddies) (Trystram et al., 2017). Two species of sharks, *Prionace glauca* (Blue Shark) and *G. cuvier*, preyed on seabirds off the coast of New South Wales, Australia. Seabirds made up 3.2% of occurrence in the stomach content of 31 *P. glauca* specimens; while 27.6% of seabird occurrence was found in the stomach contents of 29 *G. cuvier* specimens, with four seabird prey items identified as *Puffinus* spp. (shearwaters) (Stevens, 1984). In a study of the stomach contents of Black‐tip Reef Sharks, Papastamatiou et al. (2009) found that four out of 14 sampled sharks had bird remains in their stomach, including a Red‐footed Booby (*Sula sula*). Finally, Yellowtail Kingfish (*Seriola lalandi*) in New Zealand were noted to forage on Kermadec Little Shearwaters (*Puffinus assimilis kermadecensis*), fledgling Kermadec Petrels (*Pterodroma neglecta*), adult Fairy Prions (*Pachyptila turtur*), Red‐billed Gulls (*Larus novaehollandiae scopulinus*) and a Common Diving Petrel (*Pelecanoides urinatrix*) (Duffy & Taylor, 2015). These records considered as a group indicate that although poorly studied, consumption of marine birds by predatory fish is potentially important in some marine food webs, particularly where breeding colonies of birds provide a high seasonal concentration of prey.

In some tropical locations where coral reefs are present, reptiles also feed on marine birds. On Australia's Great Barrier Reef, two bands from tagged birds were recovered from Saltwater Crocodiles (*Crocodylus porosus*) at Raine Island (Dobbs, 2005) and on Milman Island, tern feathers were found inside the stomach of a Saltwater Crocodile (Dobbs et al., 1997). Dobbs et al. (1997) also mention the presence of Amethyst Pythons (*Morelia amethystina*) on Milman Island (a sandy coral cay) as generalist egg and adult bird predators, although they provide no direct records of predation on marine birds.

There are a few records of birds being taken by invertebrate predators, such as octopuses and crabs, but it is unclear how frequent these events are and there is little evidence to suggest that they play an important role in avian population dynamics. An oceanic island‐dwelling octopus (*Octopus* sp. nov.), preying on a Brown Noddy (*Anous stolidus*) in the tropical West‐Atlantic, caught the seabird while lying in ambush within a tidal pool (Sazima & Almeida, 2008). A single account of the Christmas Island Goshawk (*Accipiter hiogaster natalis*) preying on an Abbott's Booby (*Papasula abbotti*) nestling resulted in a tug of war between the Goshawk and a Robber Crab (*Birgus latro*), in which the Robber Crab ultimately secured the dead booby chick (Hennicke, 2012). Crabs and other predatory invertebrates (e.g. plough snails *Bullia* spp.; *Tritia* spp.) will also feed on marine bird carcasses.

Birds also feed on other birds; for example, Sea Eagles are frequently observed feeding on smaller marine birds and we have observed Reef Herons and Silver Gulls on Heron Island in the Great Barrier Reef regularly eating the chicks and eggs of Black Noddies. The strengths of these interactions relative to other trophic interactions (e.g. predation by sharks) have not been determined.

### 4.2 Indirect trophic effects

Birds may be involved indirectly in a wide range of interactions (Figure 1; birds may influence the magnitude of any of the effects indicated by blue arrows, through their direct or indirect effects on fishes, phytoplankton, benthic algae and corals). With the exception of nutrient enrichment, the indirect effects of birds on coral reefs are poorly understood and little‐studied, making it nearly impossible to estimate their magnitude and pointless to attempt an exhaustive or systematic review of them. Indirect interactions are both trophic (i.e. mediated through the food web) and behavioural. Indirect trophic interactions occur where birds share prey items, such as pelagic fish, with other predators. By foraging, if birds directly reduce the abundance of some species at lower trophic levels, they can indirectly reduce predation on others. For example, as depicted in Figure 1, birds may potentially influence zooplankton availability to corals by consuming pelagic fish. However, in the absence of research, the potential involvement of tropical marine birds in marine trophic cascades remains speculation.

Behavioural interactions that take place underwater influence the availability of prey items to marine birds by changing their movements and dispersion of fish prey. Of these, one of the most important is the impact of large predatory fish on schools of smaller fishes (Figure 1; arrow 9 influences arrow 2), which often move towards the surface to escape predation. Seabirds taking advantage of this effect have been described as performing ‘facilitated foraging’ (Miller et al., 2018; Spear et al., 2007). Flying fish, for example, may evade predators underwater by leaping out of the water and gliding through the air for tens of metres (Davenport, 1994), making them vulnerable to birds. This pattern is well‐documented in open oceans, where birds often feed over locations where schools of tuna and other open‐ocean fish species are foraging (Au & Pitman, 1986; Ballance et al., 1997; Jaquemet et al., 2005). In coral reef environments, foraging reef sharks and other predatory fishes may have a similar effect. For example, at Lizard Island, in the northern Great Barrier Reef, GC has observed Reef Egrets waiting on a rocky shoreline and snatching Common Hardyhead fish that were chased to the surface by small Blacktip Reef Sharks. The unpredictability and technical challenges of studying these interactions in the marine environment again mean that the behavioural interactions of predatory fishes and marine birds are poorly understood and we currently have no way of measuring their importance.

### 4.3 Indirect ecosystem effects

There are two main ways in which birds may have indirect ecosystem effects with relevance to coral reefs. The first of these is by facilitating the long‐distance dispersal of the propagules of other organisms. Although there has been relatively little research on the potential role of birds as mobile links in marine ecosystems, there is increasing evidence that freshwater birds can disperse a wide range of propagules between wetlands that are not connected by hydrological flows (Green et al., 2023). Dispersed propagules include not only plant seeds but also the eggs and other resistant life stages of invertebrates (e.g. molluscs, insects; (Reynolds & Cumming, 2015)). Marine birds in tropical locations may come into direct contact with reef‐derived propagules when bathing, drinking, courting, or resting in the water or on the shore near coral reefs. Some species exhibit additional behaviours that bring them into frequent contact with near‐shore environments, such as washing the leaves used in nest‐building (in the case of Black Noddies). Many plants found on atolls and islands have sticky seeds (e.g. *Pisonia* sp.) and birds are likely to have facilitated the colonisation of islands through both external and internal transport of seeds, with consequences for the terrestrial fauna of oceanic islands and related nutrient cycling (Burger, 2005; Walker, 1991). Birds may also in theory move the spores of algae, seagrass and other marine plants on their legs and feathers or via ingestion of seawater, although this has not been demonstrated. Heatwole and Walker (1989) suggested that as many as 22% of plant species on One Tree Island in the Great Barrier Reef and other species in the surrounding Capricorn‐Bunker area, could be dispersed by bird species such as Silver Gulls.

Second, marine birds play a key role as biological transporters of nutrients from the ocean into terrestrial habitats on islands and offshore cays. It has long been recognised that nutrients deposited in bird guano can leach in large quantities off the islands that colonial seabirds use as roosting sites and historical mining of guano islands for fertiliser was common in temperate regions (Bosman & Hockey, 1986; Duffy, 1994). Recent research in the Chagos Islands using the natural experiment of comparing islands with and without invasive rats (that impact seabird populations) has demonstrated that marine nutrients acquired and deposited by seabirds have a significant impact on the trophodynamics of coral reef communities (Benkwitt et al., 2022; Graham et al., 2018). Ecosystem effects of marine bird guano on coral reefs and adjacent habitats have also been documented across a range of additional locations (Linhares & Bugoni, 2023; Lorrain et al., 2017; Savage, 2019; Schmidt et al., 2004) and marine bird breeding colonies have been identified as key influences on nutrient flows in terrestrial and inter‐tidal food webs (Caut et al., 2012; Gagnon et al., 2013; Hentati‐Sundberg et al., 2020).

Birds are themselves indirectly influenced by reef‐building corals and island‐forming processes such as carbonate production by corals and other calcifying organisms (e.g. foraminiferans, crustose coralline algae, *Halimeda* algae) and the grazing activity of reef fishes, particularly large excavating parrotfishes (Bellwood et al., 2003; Yarlett et al., 2018) and other bioeroders. The creation of atolls and offshore coral cays and the provision of substrate that can support vegetation, offers predator‐free nesting and roosting locations for marine birds. Thus, a series of indirect feedbacks exist over geological time whereby corals create raised platforms in the ocean; excavating parrotfishes are key bioeroders capable of grinding chunks of reef into fine particulate matter that accumulates in shallow lagoons (Bellwood, 1996; Bonaldo et al., 2014), forming cays, facilitating the trapping of nutrients and the establishment and growth of vegetation and birds potentially bring in the propagules of terrestrial plants and enhance coral growth by concentrating marine nutrients in ways that enhance coral growth in the surrounding waters (Graham et al., 2018), enhancing their own habitat. The magnitudes of these relative effects are again, however, largely unknown and unmeasured in relation to birds.

## 5 GAPS AND RESEARCH NEEDS

The majority of research effort on marine birds so far has taken place in temperate regions, with tropical marine birds being widely recognised as under‐studied (Bernard et al., 2021; Orgeret et al., 2022; Oro, 2014; Sydeman et al., 2012). Despite intensive interest in marine bird ecology and a considerable amount of published research on marine birds, important gaps remain that limit our understanding of both the causes and consequences of changes in marine bird populations. These can be grouped into four main categories: (1) knowledge gaps about basic ecology; (2) knowledge gaps about direct effects; (3) knowledge gaps about indirect effects and (4) knowledge gaps about system‐wide dynamics and feedbacks in which marine birds play a role.

*Knowledge gaps about basic ecology* include information on the distributions, reproductive patterns and needs and foraging behaviours of tropical marine birds. Many tropical marine birds are poorly studied outside their breeding territories (Bernard et al., 2021; Orgeret et al., 2022) and there is little knowledge of where many species spend the non‐breeding season. Long‐distance migrants tend to be better studied, but different kinds of migration patterns or other predictable dispersal patterns are largely unexplored. In movement and foraging studies, in the same way that fish ecologists often favour studying sharks or brightly coloured tropical fishes (Bellwood et al., 2020), ornithologists frequently disregard inshore marine birds. Even within the tropical marine bird literature, Reef Egrets and other inshore species (e.g. gulls, cormorants, pelicans and oystercatchers) have largely fallen between stools; they are not treated as marine birds in most of the existing literature, but they are not typical migratory waders. Data collection for marine birds often ignores inshore species; for example, standard bird surveys conducted by Australia's Queensland Parks and Wildlife Service do not include data for Reef Egrets, but because they often live on rocky shorelines and reefs, they are often not studied as wetland birds either. Many of these species are nonetheless opportunistic, widespread predators that may be common near and on tropical coral reefs.

Individual marine bird species have different foraging styles and energetic needs and these determine the number and nature of prey items that they consume. Harrison (1983, p. 54) lists seven different foraging styles of marine birds: piracy, pattering, scavenging, dipping, plunge diving, pursuit plunging and surface seizing. He excludes additional behaviours used by inshore birds, particularly stand‐and‐wait predation and foraging in wing shadows, both of which are used extensively by Reef Egrets. There should in theory be clear relationships between foraging style and the predator–prey relationships of marine birds and marine fishes, with particular avian foraging styles favouring predation on particular taxa and sizes of fish, but these relationships have not been researched for tropical assemblages.

*Knowledge gaps about direct effects* include many research areas highlighted by this review. Our initial analysis suggests a low direct dependence of most marine bird species on coral reefs for food, but this finding will remain provisional until more evidence has been obtained. There are many more tropical marine bird species than existing data sets would suggest and published studies of the diets of many widespread and abundant tropical species found in the vicinity of coral reefs (e.g. Crested Terns, Bridled Terns and Sea Eagles) are scarce. The degree to which birds may provide top‐down population regulation or limitation of tropical fish or invertebrates has not been evaluated for any species. For example, the impact of Reef Egrets on reef flat communities, where egrets may forage at high densities on exposed benthic organisms during low tide, is unknown.

In addition to the under‐representation of avian taxa in the scientific literature, locations near coral reefs and other tropical ecosystems at which marine bird stomach contents have been sampled are concentrated in places where individual research teams are active (Figure 2a). For example, there are no studies of marine bird diets from within the Coral Triangle, the area that houses the highest diversity of coral reef fishes globally (Cowman, 2014). Even where studies of regurgitated samples of marine bird gut contents exist, the taxonomic resolution of prey items may be limited (i.e. only to the family level) likely due to the difficulty of identifying fish remains in an advanced stage of decomposition or, in some cases, limited experience in fish taxonomy to identify remains to species level. The example of the Audubon's Shearwater feeding primarily on unidentified goatfishes offers a good illustration of this problem. This may ultimately preclude a more intensive assessment of the extent of the marine bird reliance on reef fishes for food. Similarly, despite evidence (discussed above) that sharks and other large predatory fish consume marine birds, the impacts of fish predation on bird populations and reproductive success are unclear and merit further research.

*Knowledge gaps about indirect effects* are particularly apparent in relation to questions of dispersal. Many studies of tropical species assume that the primary method of dispersal for the propagules of most marine species is via oceanic currents. Evidence for birds as vectors of seeds in both temperate and tropical regions is present (Burger, 2005; Ellis, 2005), but limited; no intensive studies have been carried out. Freshwater birds, such as ducks, have been shown to successfully disperse not only plant seeds but also a range of zooplankton, invertebrates and occasionally even vertebrates (Reynolds & Cumming, 2015). Birds that move between different islands and forage, defaecate or bathe on wave‐protected areas inside reef flats could potentially carry the propagules of other species. Birds may also act as vectors of pathogens, such as influenza, that can affect other organisms, for example, recent examples of seals contracting avian influenza from seabirds (Puryear et al., 2023).

Recent research on nutrient flows from tropical marine birds has focused on cases that are amenable to study, such as locations that are far from anthropogenic nitrogen sources or where a pest‐free island can be used as a control. The effects of nitrogen additions from marine bird guano are presumably much more widespread than currently documented studies suggest and the comparative details of how marine bird colony context and heterogeneity in the abiotic environment modulate the effects of guano enrichment on tropical ecosystems are poorly understood.

*Knowledge gaps about system‐wide dynamics and feedbacks* are arguably the most glaring gap in marine bird research. The more general ecological role of marine birds and their behavioural relationships with fish, both in relation to fish influencing marine bird prey availability and marine birds influencing the success of other predators on fish, are largely a black box. Marine birds take advantage of fish that are forced to the surface by predators, as discussed above, and of discards from marine predators (Evans, 1982; Ridoux, 1987). But seabirds are also expert locators of shoals of fish and their dense foraging flocks can be visible from many miles away; humans have long used seabirds as an indicator of where to cast nets (Montevecchi, 2001) and it seems highly likely that other marine predators (seals, orcas, sharks, Minke Whales) would have learned to do the same (Anderwald et al., 2011). In the absence of any empirical evidence for or against, however, the hypothesis that tropical marine birds might act to stabilise marine food webs and/or provide some kind of buffering capacity for coral reef communities against population fluctuations of invertebrates or small pelagic fish remains purely speculative.

## 6 GENERAL DISCUSSION AND CONCLUSIONS

This first review of marine bird dependence on coral reefs suggests that although marine birds are important in many tropical marine ecosystems, most show limited direct interactions with coral reef ecosystems. The relatively weak direct benefits that reefs provide to birds may be deceptive if reefs provide a reliable but low‐level food source; reefs may contribute to the persistence of bird populations when marine conditions are unsuitable for foraging in open water. However, it seems clear that reefs alone cannot support the high densities of colonially breeding seabirds that occur on many tropical islands. Conversely, the potential relevance of birds for coral reefs appears to be primarily through indirect effects, specifically nutrient concentration and the dispersal of propagules of other species. Reef Egrets are a notable exception, where they occur on reefs, with the majority of their diet consisting of organisms captured on the reef flats.

The distance at which breeding colonies of marine birds on tropical coral islands have an impact on non‐reef marine ecosystems is unclear. Ashmole's ‘halo’ effect of seabirds on pelagic fish was only recently documented for a tropical location for the first time; a comparison of fish densities adjacent to colonies of Masked Boobies (*Sula dactylatra*) of different densities found greater food depletion near islands where bird abundance was higher (Oppel et al., 2015). The rarity of such studies limits feasible prediction of how the scale and likely magnitude of intraspecific and interspecific competition occurs in other locations.

Global marine change is already impacting the community composition and physical structure of tropical coral reefs (Alvarez‐Filip et al., 2011; Bozec et al., 2015; Hughes et al., 2018). Global marine change will also have a wide range of separate effects on food abundance for marine birds. The ability of marine birds to move from the tropics to cooler waters is constrained by coastal morphology, particularly in cases where smaller tropical species such as noddies and terns require access to suitable breeding and roosting habitats relatively close to their food supply. The separation of breeding, roosting and foraging grounds presents a high risk for colonial seabirds and may offer a point where human intervention, through the reduction of anthropogenic impacts on tropical islands and the creation of artificial structures, can help marine bird populations to persist through the period of change that we are currently entering. Ultimately, we conclude that marine birds are more likely to be victims of broader‐scale shifts in marine ecosystems than casualties of coral reef degradation.

## AUTHOR CONTRIBUTIONS

**Graeme S. Cumming:** Conceptualization (lead); data curation (supporting); formal analysis (lead); funding acquisition (lead); investigation (lead); methodology (lead); project administration (lead); supervision (lead); visualization (supporting); writing – original draft (lead); writing – review and editing (lead). **Nicholas L. James:** Writing – review and editing (supporting). **Chia Miin Chua:** Data curation (supporting); investigation (supporting); writing – review and editing (supporting). **Victor Huertas:** Conceptualization (supporting); data curation (lead); formal analysis (equal); investigation (supporting); methodology (equal); software (lead); validation (lead); visualization (lead); writing – original draft (supporting); writing – review and editing (supporting).

## FUNDING INFORMATION

This research was funded by the ARC Centre of Excellence for Coral Reef Studies, James Cook University, the University of Western Australia and a Western Australian Premier's Science Fellowship awarded to GC.

## Supporting information


Appendix S1

Appendix S2

Appendix S3

## Data Availability

All data used in the article are provided in the Appendices that were uploaded with this file.

## References

[ece370165-bib-0001] Ahyong, S. , Boyko, C. B. , Bailly, N. , Bernot, J. , Bieler, R. , Brandão, S. N. , Daly, M. , De Grave, S. , Gofas, S. , Hernandez, F. , Hughes, L. , Neubauer, T. A. , Paulay, G. , Boydens, B. , Decock, W. , Dekeyzer, S. , Vandepitte, L. , Vanhoorne, B. , Adlard, R. , … Zullini, A. (2024). World register of marine species (WoRMS). WoRMS Editorial Board. https://www.marinespecies.org

[ece370165-bib-0002] Ainley, D. G. , Walker, W. A. , Spencer, G. C. , & Holmes, N. D. (2014). The prey of Newell's shearwater *Puffinus newelli* in Hawaiian waters. Marine Ornithology, 44, 69–72.

[ece370165-bib-0003] Ainsworth, T. D. , Heron, S. F. , Ortiz, J. C. , Mumby, P. J. , Grech, A. , Ogawa, D. , Eakin, C. M. , & Leggat, W. P. (2016). Climate change disables coral bleaching protection on the great barrier reef. Science, 352(11), 338–342. 10.1126/science.aac7125 27081069

[ece370165-bib-0004] Alabia, I. D. , Saitoh, S.‐I. , Igarashi, H. , Ishikawa, Y. , Usui, N. , Kamachi, M. , Awaji, T. , & Seito, M. (2016). Future projected impacts of ocean warming to potential squid habitat in western and central North Pacific. ICES Journal of Marine Science, 73(5), 1343–1356. 10.1093/icesjms/fsv203

[ece370165-bib-0005] Alvarez‐Filip, L. , Côté, I. M. , Gill, J. A. , Watkinson, A. R. , & Dulvy, N. K. (2011). Region‐wide temporal and spatial variation in Caribbean reef architecture: Is coral cover the whole story? Global Change Biology, 17(7), 2470–2477. 10.1111/j.1365-2486.2010.02385.x

[ece370165-bib-0006] Alves, V. S. , Soares, A. B. A. , Couto, G. S. D. , Efe, M. A. , & Ribeiro, A. B. B. (2004). Aves Marinhas de Abrolhos—Bahia, Brasil. In J. O. Branco (Ed.), Aves marinhas e insulares Brasileiras: Bioecologia e conservacao (pp. 213–232). Editora da UNIVALI.

[ece370165-bib-0007] Anderson, W. B. , & Polis, G. A. (1999). Nutrient fluxes from water to land: Seabirds affect plant nutrient status on gulf of California islands. Oecologia, 118, 324–332. 28307276 10.1007/s004420050733

[ece370165-bib-0008] Anderwald, P. , Evans, P. G. , Gygax, L. , & Hoelzel, A. R. (2011). Role of feeding strategies in seabird–minke whale associations. Marine Ecology Progress Series, 424, 219–227.

[ece370165-bib-0009] Aoyama, Y. , Kawakami, K. , & Chiba, S. (2012). Seabirds as adhesive seed dispersers of alien and native plants in the oceanic Ogasawara Islands, Japan. Biodiversity and Conservation, 21(11), 2787–2801. 10.1007/s10531-012-0336-9

[ece370165-bib-0010] Arkhipkin, A. I. , Rodhouse, P. G. , Pierce, G. J. , Sauer, W. , Sakai, M. , Allcock, L. , Arguelles, J. , Bower, J. R. , Castillo, G. , & Ceriola, L. (2015). World squid fisheries. Reviews in Fisheries Science & Aquaculture, 23(2), 92–252. 10.1080/23308249.2015.1026226

[ece370165-bib-0011] Ashmole, N. P. , & Ashmole, M. J. (1967). Comparative feeding ecology of sea birds of a tropical Oceanic Island (Bulletin 24; Peabody Museum of Natural History, p. 75). Yale University. 10.2307/4511544?origin=crossref

[ece370165-bib-0012] Asseid, B. S. , Drapeau, L. , Crawford, R. J. M. , Dyer, B. M. , Hija, A. , Mwinyi, A. , Shinula, P. , & Upfold, L. (2006). The food of three seabirds at Latham Island, Tanzania, with observations on foraging by masked boobies *Sula dactylatra* . African Journal of Marine Science, 28(1), 109–114. 10.2989/18142320609504138

[ece370165-bib-0013] Au, D. W. K. , & Pitman, R. L. (1986). Seabird interactions with dolphins and tuna in the eastern tropical Pacific. The Condor, 88(3), 304–317. 10.2307/1368877

[ece370165-bib-0014] Austin, R. E. , De Pascalis, F. , Votier, S. C. , Haakonsson, J. , Arnould, J. P. Y. , Ebanks‐Petrie, G. , Newton, J. , Harvey, J. , & Green, J. A. (2021). Interspecific and intraspecific foraging differentiation of neighbouring tropical seabirds. Movement Ecology, 9(1), 27. 10.1186/s40462-021-00251-z 34039419 PMC8152358

[ece370165-bib-0015] Austin, R. E. , Pascalis, F. , Arnould, J. , Haakonsson, J. , Votier, S. , Ebanks‐Petrie, G. , Austin, T. , Morgan, G. , Bennett, G. , & Green, J. (2019). A sex‐influenced flexible foraging strategy in a tropical seabird, the magnificent frigatebird. Marine Ecology Progress Series, 611, 203–214. 10.3354/meps12859

[ece370165-bib-0016] Ballance, L. T. , Pitman, R. L. , & Reilly, S. B. (1997). Seabird community structure along a productivity gradient: Importance of competition and energetic constraint. Ecology, 78(5), 1502–1518. 10.1890/0012-9658(1997)078[1502:SCSAAP]2.0.CO;2

[ece370165-bib-0017] Baum, J. K. , & Worm, B. (2009). Cascading top‐down effects of changing oceanic predator abundances. Journal of Animal Ecology, 78(4), 699–714. 10.1111/j.1365-2656.2009.01531.x 19298616

[ece370165-bib-0018] Bellwood, D. R. (1996). Production and reworking of sediment by parrotfishes (family Scaridae) on the Great Barrier Reef, Australia. Marine Biology, 125, 795–800.

[ece370165-bib-0019] Bellwood, D. R. , Hemingson, C. R. , & Tebbett, S. B. (2020). Subconscious biases in coral reef fish studies. Bioscience, 70(7), 621–627. 10.1093/biosci/biaa062

[ece370165-bib-0020] Bellwood, D. R. , Hoey, A. S. , & Choat, J. H. (2003). Limited functional redundancy in high diversity systems: Resilience and ecosystem function of coral reefs. Ecology Letters, 6, 281–285. 10.1046/j.1461-0248.2003.00432.x

[ece370165-bib-0021] Bellwood, D. R. , & Wainwright, P. C. (2002). The history and biogeography of fishes on coral reefs. Coral Reef Fishes: Dynamics and Diversity in a Complex Ecosystem, 5, 32.

[ece370165-bib-0022] Benkwitt, C. E. , Carr, P. , Wilson, S. K. , & Graham, N. A. (2022). Seabird diversity and biomass enhance cross‐ecosystem nutrient subsidies. Proceedings of the Royal Society B, 289(1974), 20220195. 35538790 10.1098/rspb.2022.0195 PMC9091852

[ece370165-bib-0023] Bernard, A. , Rodrigues, A. S. L. , Cazalis, V. , & Grémillet, D. (2021). Toward a global strategy for seabird tracking. Conservation Letters, 14(3), e12804. 10.1111/conl.12804

[ece370165-bib-0024] Bester, A. J. , Priddel, D. , & Klomp, N. I. (2011). Diet and foraging behaviour of the providence petrel *Pterodroma solandri* . Marine Ornithology, 39, 163–172.

[ece370165-bib-0025] Blaber, S. , Milton, D. , Smith, G. , & Farmer, M. (1995). Trawl discards in the diets of tropical seabirds of the northern great barrier reef, Australia. Marine Ecology Progress Series, 127, 1–13. 10.3354/meps127001

[ece370165-bib-0026] Blaber, S. , & Wassenberg, T. J. (1989). Feeding ecology of the piscivorous birds *Phalacrocorax varius*, *P. melanoleucos* and *Sterna bergii* in Moreton Bay, Australia: Diets and dependence on trawler discards. Marine Biology, 101, 1–10. 10.1007/BF00393473

[ece370165-bib-0027] Boettiger, C. , Lang, D. T. , & Wainwright, P. C. (2012). Rfishbase: Exploring, manipulating and visualizing FishBase data from R. Journal of Fish Biology, 81(6), 2030–2039. 10.1111/j.1095-8649.2012.03464.x 23130696

[ece370165-bib-0028] Bonaldo, R. M. , Hoey, A. S. , & Bellwood, D. R. (2014). The ecosystem roles of parrotfishes on tropical reefs. Oceanography and Marine Biology: An Annual Review, 52, 81–132.

[ece370165-bib-0029] Bosman, A. L. , & Hockey, P. A. R. (1986). Seabird guano as a determinant of rocky intertidal community structure. Marine Ecology Progress Series, 32, 247–257.

[ece370165-bib-0030] Bozec, Y.‐M. , Alvarez‐Filip, L. , & Mumby, P. J. (2015). The dynamics of architectural complexity on coral reefs under climate change. Global Change Biology, 21(1), 223–235. 10.1111/gcb.12698 25099220

[ece370165-bib-0031] Brown, W. Y. (1975). Parental feeding of young sooty terns (*Sterna fuscata* (L.)) and Brown Noddies (*Anous stolidus* (L.)) in Hawaii. The Journal of Animal Ecology, 44(3), 731. 10.2307/3715

[ece370165-bib-0032] Burger, A. E. (2005). Dispersal and germination of seeds of *Pisonia grandis*, an indo‐Pacific tropical tree associated with insular seabird colonies. Journal of Tropical Ecology, 21(3), 263–271.

[ece370165-bib-0033] Catry, T. , Ramos, J. , Jaquemet, S. , Faulquier, L. , Berlincourt, M. , Hauselmann, A. , Pinet, P. , & Le Corre, M. (2009). Comparative foraging ecology of a tropical seabird community of the Seychelles, western Indian Ocean. Marine Ecology Progress Series, 374, 259–272. 10.3354/meps07713

[ece370165-bib-0034] Caut, S. , Angulo, E. , Pisanu, B. , Ruffino, L. , Faulquier, L. , Lorvelec, O. , Chapuis, J.‐L. , Pascal, M. , Vidal, E. , & Courchamp, F. (2012). Seabird modulations of isotopic nitrogen on islands. PLoS One, 7(6), e39125. 10.1371/journal.pone.0039125 22723945 PMC3377609

[ece370165-bib-0035] Chamberlain, S. (2020). *worrms: World register of marine species (WoRMS) client* (0.4.2) [Computer software]. https://CRAN.R‐project.org/package=worrms

[ece370165-bib-0036] Chiang, C.‐I. , Chung, M.‐T. , Shiao, J.‐C. , Wang, P.‐L. , Chan, T.‐Y. , Yamaguchi, A. , & Wang, C.‐H. (2020). Seasonal movement patterns of the bigfin reef squid *Sepioteuthis lessoniana* predicted using statolith δ18O values. Frontiers in Marine Science, 7, 249. 10.3389/fmars.2020.00249

[ece370165-bib-0037] Correia, E. , Catry, P. , Sinclair, F. , Dos Santos, Y. , Robalo, J. I. , Lima, C. S. , & Granadeiro, J. P. (2021). Foraging behaviour and diet of Brown boobies *Sula leucogaster* from Tinhosas Islands, Gulf of Guinea. Marine Biology, 168(6), 91. 10.1007/s00227-021-03904-0

[ece370165-bib-0038] Cowman, P. F. (2014). Historical factors that have shaped the evolution of tropical reef fishes: A review of phylogenies, biogeography, and remaining questions. Frontiers in Genetics, 5, 394. 10.3389/fgene.2014.00394 25431581 PMC4230204

[ece370165-bib-0039] Croxall, J. P. , Butchart, S. H. , Lascelles, B. E. N. , Stattersfield, A. J. , Sullivan, B. E. N. , Symes, A. , & Taylor, P. (2012). Seabird conservation status, threats and priority actions: A global assessment. Bird Conservation International, 22(1), 1–34.

[ece370165-bib-0040] Davenport, J. (1994). How and why do flying fish fly? Reviews in Fish Biology and Fisheries, 4(2), 184–214. 10.1007/BF00044128

[ece370165-bib-0041] Diamond, A. W. (1974). The red‐footed booby on Aldabra Atoll, Indian Ocean. Ardea, 62, 196–218.

[ece370165-bib-0042] Diamond, A. W. (1975a). Biology and behaviour of Frigatebirds *Fregata* spp. on Aldabra Atoll. Ibis, 117, 302–323. 10.1111/j.1474-919X.1975.tb04219.x

[ece370165-bib-0043] Diamond, A. W. (1975b). The biology of tropicbirds at Aldabra Atoll, Indian Ocean. The Auk, 92(1), 16–39. 10.2307/4084415

[ece370165-bib-0044] Diamond, A. W. (1983). Feeding overlap in some tropical and temperate seabird communities. Studies in Avian Biology, 8, 24–46.

[ece370165-bib-0045] Dias, M. P. , Martin, R. , Pearmain, E. J. , Burfield, I. J. , Small, C. , Phillips, R. A. , Yates, O. , Lascelles, B. , Borboroglu, P. G. , & Croxall, J. P. (2019). Threats to seabirds: A global assessment. Biological Conservation, 237, 525–537.

[ece370165-bib-0046] Dobbs, K. A. (2005). Recoveries of seabirds banded between 1978 and 1987 at Raine Island, MacLennan and Moulter cays and sandbanks No. 7 and 8, northern great barrier reef, Australia. Corella, 29(3), 65.

[ece370165-bib-0047] Dobbs, K. A. , Miller, J. D. , Card, M. A. , Mather, M. , & Haselmayer, J. (1997). Birds of Milman Island. Corella, 21, 37–43.

[ece370165-bib-0048] Donahue, S. E. , Adams, J. , Renshaw, M. A. , & Hyrenbach, K. D. (2021). Genetic analysis of the diet of red‐footed boobies (*Sula sula*) provisioning chicks at Ulupa'u crater, O'ahu. Aquatic Conservation: Marine and Freshwater Ecosystems, 31(2), 324–339. 10.1002/aqc.3470

[ece370165-bib-0049] Duffy, C. A. J. , & Taylor, G. A. (2015). Predation on seabirds by large teleost fishes in northern New Zealand. Bulletin of the Aukland Museum, 20, 497–500.

[ece370165-bib-0050] Duffy, D. C. (1994). The guano islands of Peru: The once and future management of a renewable resource. Birdlife Conservation Series, 1, 68–76.

[ece370165-bib-0051] Ellis, J. C. (2005). Marine birds on land: A review of plant biomass, species richness, and community composition in seabird colonies. Plant Ecology, 181(2), 227–241. 10.1007/s11258-005-7147-y

[ece370165-bib-0052] Evans, P. G. H. (1982). Associations between seabirds and cetaceans: A review. Mammal Review, 12(4), 187–206.

[ece370165-bib-0053] Froese, R. , & Pauly, D. (2023). FishBase. World Wide Web Electornic Publication. www.fishbase.org

[ece370165-bib-0054] Gagnon, K. , Rothäusler, E. , Syrjänen, A. , Yli‐Renko, M. , & Jormalainen, V. (2013). Seabird guano fertilizes Baltic Sea Littoral food webs. PLoS One, 8(4), e61284. 10.1371/journal.pone.0061284 23593452 PMC3623859

[ece370165-bib-0055] Gallagher, A. J. , Jackson, T. , & Hammerschlag, N. (2011). Occurrence of tiger shark (*Galeocerdo cuvier*) scavenging on avian prey and its possible connection to large‐scale bird die‐offs in the Florida keys. Florida Scientist, 74(4), 264–269.

[ece370165-bib-0056] Graham, N. A. J. , Wilson, S. K. , Carr, P. , Hoey, A. S. , Jennings, S. , & MacNeil, M. A. (2018). Seabirds enhance coral reef productivity and functioning in the absence of invasive rats. Nature, 559(7713), 250–253. 10.1038/s41586-018-0202-3 29995864

[ece370165-bib-0057] Grant, M. L. , Bond, A. L. , & Lavers, J. L. (2022). The influence of seabirds on their breeding, roosting and nesting grounds: A systematic review and meta‐analysis. Journal of Animal Ecology, 91(6), 1266–1289. 35395097 10.1111/1365-2656.13699 PMC9324971

[ece370165-bib-0058] Green, A. J. , Lovas‐Kiss, Á. , Reynolds, C. , Sebastián‐González, E. , Silva, G. G. , van Leeuwen, C. H. , & Wilkinson, D. M. (2023). Dispersal of aquatic and terrestrial organisms by waterbirds: A review of current knowledge and future priorities. Freshwater Biology, 68(2), 173–190.

[ece370165-bib-0059] Harrison, C. S. , Hida, T. S. , & Seki, M. P. (1983). Hawaiian seabird feeding ecology. Wildlife Monographs, 85, 1–71.

[ece370165-bib-0060] Harrison, C. S. , Hida, T. S. , & Seki, M. P. (1984). The diet of the Brown booby *Sula leucogaster* and masked booby *Sula dactylatra* on rose atoll, Samoa. Ibis, 126(4), 588–590. 10.1111/j.1474-919X.1984.tb02082.x

[ece370165-bib-0061] Hays, G. C. , Koldewey, H. J. , Andrzejaczek, S. , Attrill, M. J. , Barley, S. , Bayley, D. T. , Benkwitt, C. E. , Block, B. , Schallert, R. J. , & Carlisle, A. B. (2020). A review of a decade of lessons from one of the world's largest MPAs: Conservation gains and key challenges. Marine Biology, 167, 1–22. 10.1007/s00227-020-03776-w

[ece370165-bib-0062] Heatwole, H. , & Walker, T. A. (1989). Dispersal of alien plants to coral cays. Ecology, 70(3), 787–790.

[ece370165-bib-0063] Heithaus, M. R. , Wirsing, A. J. , Burkholder, D. , Thomson, J. , & Dill, L. M. (2009). Towards a predictive framework for predator risk effects: The interaction of landscape features and prey escape tactics. Journal of Animal Ecology, 78(3), 556–562. 10.1111/j.1365-2656.2008.01512.x 19076259

[ece370165-bib-0064] Hennicke, J. C. (2012). Christmas Island goshawk predation on abbott's booby chick and competition with a robber crab. Australian Field Ornithology, 29(4), 196–200.

[ece370165-bib-0065] Hentati‐Sundberg, J. , Raymond, C. , Sköld, M. , Svensson, O. , Gustafsson, B. , & Bonaglia, S. (2020). Fueling of a marine‐terrestrial ecosystem by a major seabird colony. Scientific Reports, 10(1), 15455. 10.1038/s41598-020-72238-6 32963305 PMC7508978

[ece370165-bib-0066] Hughes, T. P. , Kerry, J. T. , Álvarez‐Noriega, M. , Álvarez‐Romero, J. , Anderson, K. D. , Baird, A. H. , Babcock, R. C. , Beger, M. , Bellwood, D. R. , Berkelmans, R. , Bridge, T. C. L. , Butler, I. , Byrne, M. , Cantin, N. , Comeau, S. , Connolly, S. R. , Cumming, G. S. , Dalton, S. J. , Diaz‐Pulido, G. , … Wilson, S. K. (2017). Global warming and recurrent mass bleaching of corals. Nature, 543(7645), 373–378. 10.1038/nature21707 28300113

[ece370165-bib-0067] Hughes, T. P. , Kerry, J. T. , Baird, A. H. , Connolly, S. R. , Dietzel, A. , Eakin, M. , Heron, S. F. , Hoey, A. S. , Hoogenboom, M. O. , Liu, G. , McWilliam, M. J. , Pears, R. J. , Pratchett, M. S. , Skirving, W. J. , Stella, J. S. , & Torda, G. (2018). Global warming transforms coral reef assemblages. Nature, 556, 492–496. 10.1038/s41586-018-0041-2 29670282

[ece370165-bib-0130] James, N. L. , & Cumming, G. S. (2024). Climate change may impact habitat complementation and cause disassociation for mobile species. Landscape Ecology, 39, 139.

[ece370165-bib-0068] Jaquemet, S. , Le Corre, M. , Marsac, F. , Potier, M. , & Weimerskirch, H. (2005). Foraging habitats of the seabird community of Europa Island (Mozambique Channel). Marine Biology, 147(3), 573–582. 10.1007/s00227-005-1610-0

[ece370165-bib-0069] Jaquemet, S. , Potier, M. , Cherel, Y. , Kojadinovic, J. , Bustamante, P. , Richard, P. , Catry, T. , Ramos, J. A. , & Le Corre, M. (2008). Comparative foraging ecology and ecological niche of a superabundant tropical seabird: The sooty tern *Sterna fuscata* in the southwest Indian Ocean. Marine Biology, 155(5), 505–520. 10.1007/s00227-008-1049-1

[ece370165-bib-0070] Jones, C. J. , Lyver, P. O. , Davis, J. , Hughes, B. , Anderson, A. , & Hohapata‐Oke, J. (2015). Reinstatement of customary seabird harvests after a 50‐year moratorium: Reinstating seabird harvest on islands. The Journal of Wildlife Management, 79(1), 31–38. 10.1002/jwmg.815

[ece370165-bib-0071] Le Corre, M. , Cherel, Y. , Lagarde, F. , Lormée, H. , & Jouventin, P. (2003). Seasonal and inter‐annual variation in the feeding ecology of a tropical oceanic seabird, the red‐tailed tropicbird *Phaethon rubricauda* . Marine Ecology Progress Series, 255, 289–301. 10.3354/meps255289

[ece370165-bib-0072] Le Corre, M. , Jaeger, A. , Pinet, P. , Kappes, M. A. , Weimerskirch, H. , Catry, T. , Ramos, J. A. , Russell, J. C. , Shah, N. , & Jaquemet, S. (2012). Tracking seabirds to identify potential marine protected areas in the tropical western Indian Ocean. Biological Conservation, 156, 83–93. 10.1016/j.biocon.2011.11.015

[ece370165-bib-0073] Leis, J. M. , & McCormick, M. I. (2002). The biology, behavior, and ecology of the pelagic, larval stage of coral reef fishes. In P. F. Sale (Ed.), Coral reef fishes (pp. 171–199). Elsevier. 10.1016/B978-012615185-5/50011-6

[ece370165-bib-0074] Lepage, D. , Vaidya, G. , & Guralnick, R. (2014). Avibase – A database system for managing and organizing taxonomic concepts. ZooKeys, 420, 117–135. 10.3897/zookeys.420.7089 PMC4109484 25061375

[ece370165-bib-0075] Linhares, B. d. A. , & Bugoni, L. (2023). Seabirds subsidize terrestrial food webs and coral reefs in a tropical rat‐invaded archipelago. Ecological Applications, 33(2), e2733. 36057541 10.1002/eap.2733

[ece370165-bib-0076] Lorrain, A. , Houlbrèque, F. , Benzoni, F. , Barjon, L. , Tremblay‐Boyer, L. , Menkes, C. , Gillikin, D. P. , Payri, C. , Jourdan, H. , Boussarie, G. , Verheyden, A. , & Vidal, E. (2017). Seabirds supply nitrogen to reef‐building corals on remote Pacific islets. Scientific Reports, 7(1), 3721. 10.1038/s41598-017-03781-y 28623288 PMC5473863

[ece370165-bib-0077] Madden, H. , Boehm, H. , & Mielke, L. (2023). Foraging ecology of red‐billed tropicbirds on Saba, Caribbean Netherlands, during early chick‐rearing. Ardea, 111(2), 463–475. 10.5253/arde.2022.a14

[ece370165-bib-0078] Madden, H. , Satgé, Y. , Wilkinson, B. , & Jodice, P. G. R. (2022). Foraging ecology of red‐billed tropicbird *Phaethon aethereus* in the Caribbean during early chick rearing revealed by GPS tracking. Marine Ornithology, 50, 165–175.

[ece370165-bib-0079] McDuie, F. , & Congdon, B. (2016). Trans‐equatorial migration and non‐breeding habitat of tropical shearwaters: Implications for modelling pelagic important bird areas. Marine Ecology Progress Series, 550, 219–234. 10.3354/meps11713

[ece370165-bib-0080] McDuie, F. , Weeks, S. J. , & Congdon, B. C. (2018). Oceanographic drivers of near‐colony seabird foraging site use in tropical marine systems. Marine Ecology Progress Series, 589, 209–225. 10.3354/meps12475

[ece370165-bib-0081] McDuie, F. , Weeks, S. J. , Miller, M. G. R. , & Congdon, B. C. (2015). Breeding tropical shearwaters use distant foraging sites when self‐provisioning. Marine Ornithology, 43, 123–129.

[ece370165-bib-0082] Meyer, C. G. , Papastamatiou, Y. P. , & Holland, K. N. (2010). A multiple instrument approach to quantifying the movement patterns and habitat use of tiger (*Galeocerdo cuvier*) and Galapagos sharks (*Carcharhinus galapagensis*) at French frigate shoals, Hawaii. Marine Biology, 157(8), 1857–1868. 10.1007/s00227-010-1457-x

[ece370165-bib-0083] Miller, M. G. , Carlile, N. , Phillips, J. S. , McDuie, F. , & Congdon, B. C. (2018). Importance of tropical tuna for seabird foraging over a marine productivity gradient. Marine Ecology Progress Series, 586, 233–249.

[ece370165-bib-0084] Miller, M. J. (2009). Ecology of anguilliform leptocephali: Remarkable transparent fish larvae of the ocean surface layer. Aqua‐BioScience Monographs, 2(4), 1–94. 10.5047/absm.2009.00204.0001

[ece370165-bib-0085] Moller, H. , O'Blyver, P. , Bragg, C. , Newman, J. , Clucas, R. , Fletcher, D. , Kitson, J. , McKechnie, S. , Scott, D. , & Rakiura Titi Islands Administering . (2009). Guidelines for cross‐cultural participatory action research partnerships: A case study of a customary seabird harvest in New Zealand. New Zealand Journal of Zoology, 36(3), 211–241. 10.1080/03014220909510152

[ece370165-bib-0086] Montevecchi, W. A. (2001). Interactions between fisheries and seabirds. In E. A. Schreiber & J. Burger (Eds.), Biology of marine birds (pp. 545–576). CRC Press.

[ece370165-bib-0087] Monticelli, D. , Ramos, J. A. , Tavares, P. C. , Bataille, B. , Lepoint, G. , & Devillers, P. (2008). Diet and foraging ecology of roseate terns and lesser Noddies breeding Sympatrically on Aride Island, Seychelles. Waterbirds, 31(2), 231–240. 10.1675/1524-4695(2008)31[231:DAFEOR]2.0.CO;2

[ece370165-bib-0088] Nascimento, I. L. S. , & Azevedo‐Junior, S. M. (2005). Dieta das Aves Marinhas no Parque Nacional dos Abrolhos, Bahia, Brasil. Ornithologia, 1(1), 75–92.

[ece370165-bib-0089] O'Dor, R. , Adamo, S. , Aitken, J. , Andrade, Y. , Finn, J. , Hanlon, R. , & Jackson, G. (2002). Currents as environmental constraints on the behavior, energetics and distribution of squid and cuttlefish. Bulletin of Marine Science, 71(2), 601–617.

[ece370165-bib-0090] Oppel, S. , Beard, A. , Fox, D. , Mackley, E. , Leat, E. , Henry, L. , Clingham, E. , Fowler, N. , Sim, J. , Sommerfeld, J. , Weber, N. , Weber, S. , & Bolton, M. (2015). Foraging distribution of a tropical seabird supports Ashmole's hypothesis of population regulation. Behavioral Ecology and Sociobiology, 69(6), 915–926. 10.1007/s00265-015-1903-3

[ece370165-bib-0091] Orgeret, F. , Thiebault, A. , Kovacs, K. M. , Lydersen, C. , Hindell, M. A. , Thompson, S. A. , Sydeman, W. J. , & Pistorius, P. A. (2022). Climate change impacts on seabirds and marine mammals: The importance of study duration, thermal tolerance and generation time. Ecology Letters, 25(1), 218–239. 10.1111/ele.13920 34761516

[ece370165-bib-0092] Oro, D. (2014). Seabirds and climate: Knowledge, pitfalls, and opportunities. Frontiers in Ecology and Evolution, 2, Article 79. 10.3389/fevo.2014.00079

[ece370165-bib-0093] Papastamatiou, Y. P. , Caselle, J. E. , Friedlander, A. M. , & Lowe, C. G. (2009). Distribution, size frequency, and sex ratios of blacktip reef sharks *Carcharhinus melanopterus* at Palmyra atoll: A predator‐dominated ecosystem. Journal of Fish Biology, 75(3), 647–654. 20738562 10.1111/j.1095-8649.2009.02329.x

[ece370165-bib-0094] Parsons, M. , Mitchell, I. , Butler, A. , Ratcliffe, N. , Frederiksen, M. , Foster, S. , & Reid, J. B. (2008). Seabirds as indicators of the marine environment. ICES Journal of Marine Science, 65(8), 1520–1526.

[ece370165-bib-0095] Pinet, P. , Jaquemet, S. , Pinaud, D. , Weimerskirch, H. , Phillips, R. , & Le Corre, M. (2011). Migration, wintering distribution and habitat use of an endangered tropical seabird, Barau's petrel *Pterodroma baraui* . Marine Ecology Progress Series, 423, 291–302. 10.3354/meps08971

[ece370165-bib-0096] Polovina, J. J. (1984). Model of a coral reef ecosystem. 1. The ECOPATH model and its application to French frigate shoals. Coral Reefs, 3, 1–11.

[ece370165-bib-0097] Puryear, W. , Sawatzki, K. , Hill, N. , Foss, A. , Stone, J. J. , Doughty, L. , Walk, D. , Gilbert, K. , Murray, M. , & Cox, E. (2023). Highly pathogenic avian influenza a (H5N1) virus outbreak in New England seals, United States. Emerging Infectious Diseases, 29(4), 786–791. 36958010 10.3201/eid2904.221538 PMC10045683

[ece370165-bib-0098] Recher, H. F. (1972). Territorial and agonistic behaviour of the reef Heron. Emu, 72(4), 126–130. 10.1071/mu972126

[ece370165-bib-0099] Recher, H. F. , & Recher, J. A. (1972). The foraging behaviour of the reef Heron. Emu, 72(3), 85–90. 10.1071/mu972085

[ece370165-bib-0100] Reynolds, C. , & Cumming, G. S. (2015). The role of waterbirds in the dispersal of freshwater cladocera and bryozoa in southern Africa. African Zoology, 50(4), 307–311.

[ece370165-bib-0101] Ridoux, V. (1987). Feeding association between seabirds and killer whales, *Orcinus orca*, around subantarctic Crozet Islands. Canadian Journal of Zoology, 65(8), 2113–2115.

[ece370165-bib-0102] Rohwer, S. (1998). Foraging differences between white and dark morphs of the Pacific reef Heron *Egretta sacra* . Ibis, 132(1), 21–26. 10.1111/j.1474-919X.1990.tb01012.x

[ece370165-bib-0103] Savage, C. (2019). Seabird nutrients are assimilated by corals and enhance coral growth rates. Scientific Reports, 9(1), 4284. 30862902 10.1038/s41598-019-41030-6 PMC6414626

[ece370165-bib-0104] Sazima, I. , & Almeida, L. B. (2008). The bird kraken: Octopus preys on a sea bird at an oceanic Island in the tropical West Atlantic. Marine Biodiversity Records, 1, 47. 10.1017/S1755267206005458

[ece370165-bib-0105] Schmidt, S. , Dennison, W. C. , Moss, G. J. , & Stewart, G. R. (2004). Nitrogen ecophysiology of Heron Island, a subtropical coral cay of the great barrier reef, Australia. Functional Plant Biology, 31(5), 517–528. 32688923 10.1071/FP04024

[ece370165-bib-0106] Schreiber, R. W. , & Hensley, D. A. (1976). The diets of *Sula dactylatra*, *Sula sula*, and *Fregata minor* on Christmas Island, Pacific Ocean. Pacific Science, 30(3), 241–248.

[ece370165-bib-0107] Seki, M. P. , & Harrison, C. S. (1989). Feeding ecology of two subtropical seabird species at French frigate shoals, Hawaii. Bulletin of Marine Science, 45(1), 52–67.

[ece370165-bib-0108] Shealer, D. A. (1998). Differences in diet and Chick provisioning between adult roseate and Sandwich terns in Puerto Rico. The Condor, 100(1), 131–140. 10.2307/1369904

[ece370165-bib-0109] Sherley, R. B. , Ladd‐Jones, H. , Garthe, S. , Stevenson, O. , & Votier, S. C. (2020). Scavenger communities and fisheries waste: North Sea discards support 3 million seabirds, 2 million fewer than in 1990. Fish and Fisheries, 21(1), 132–145.

[ece370165-bib-0110] Simpfendorfer, C. A. (1992). Biology of tiger sharks *Galeocerdo cuvier* caught by the Queensland shark meshing program off Townsville, Australia. Marine and Freshwater Research, 43(1), 33–43. 10.1071/MF9920033

[ece370165-bib-0111] Simpfendorfer, C. A. , Goodreid, A. B. , & McAuley, R. B. (2001). Size, sex and geographic variation in the diet of the tiger shark, *Galeocerdo cuvier*, from western australian waters. Environmental Biology of Fishes, 61(1), 37–46. 10.1023/A:1011021710183

[ece370165-bib-0112] Siqueira, A. C. , Morais, R. A. , Bellwood, D. R. , & Cowman, P. F. (2020). Trophic innovations fuel reef fish diversification. Nature Communications, 11(1), 2669. 10.1038/s41467-020-16498-w PMC7260216 32472063

[ece370165-bib-0113] Smith, G. C. (1985). An analysis of prey remnants from osprey *Pandion haliaetus* and white‐Bellied Sea‐eagle *Haliaetus leucogaster* feeding roosts. Emu, 85(3), 198–200. 10.1071/MU9850198

[ece370165-bib-0114] Smith, G. C. (1990). Factors influencing egg laying and feeding in black‐naped terns *Sterna sumatrana* . Emu, 90(2), 88–96. 10.1071/MU9900088

[ece370165-bib-0115] Smith, G. C. (1993). Feeding and breeding of crested terns at a tropical locality—Comparison with sympatric black‐naped terns. Emu, 93(2), 65–70. 10.1071/MU9930065

[ece370165-bib-0116] Spear, L. B. , Ainley, D. G. , & Walker, W. A. (2007). Foraging dynamics of seabirds in the eastern tropical Pacific Ocean. Cooper Ornithological Society.

[ece370165-bib-0117] Stevens, J. D. (1984). Biological observations on sharks caught by sport fisherman of New South Wales. Marine and Freshwater Research, 35(5), 573–590. 10.1071/MF9840573

[ece370165-bib-0118] Stevens, J. D. , & McLoughlin, K. J. (1991). Distribution, size and sex composition, reproductive biology and diet of sharks from northern Australia. Australian Journal of Marine and Freshwater Research, 42(2), 151–199. 10.1071/MF9910151

[ece370165-bib-0119] Surman, C. A. , & Wooller, R. D. (2003). Comparative foraging ecology of five sympatric terns at a sub‐tropical Island in the eastern Indian Ocean. Journal of Zoological Society of London, 259(3), 219–230. 10.1017/S0952836902003047

[ece370165-bib-0120] Sydeman, W. J. , Thompson, S. A. , & Kitaysky, A. (2012). Seabirds and climate change: Roadmap for the future. Marine Ecology Progress Series, 454, 107–117. 10.3354/meps09806

[ece370165-bib-0121] Tayefeh, F. H. , Zakaria, M. , Amini, H. , Mohammadnejad, J. , Darvishi, K. , & Karami, S. (2014). Dietary segregation between breeding tern species on the Persian Gulf Islands, Iran. Waterbirds, 37(3), 307–318. 10.1675/063.037.0309

[ece370165-bib-0122] Trevail, A. M. , Nicoll, M. A. , Freeman, R. , Le Corre, M. , Schwarz, J. , Jaeger, A. , Bretagnolle, V. , Calabrese, L. , Feare, C. , & Lebarbenchon, C. (2023). Tracking seabird migration in the tropical Indian Ocean reveals basin‐scale conservation need. Current Biology, 33(23), 5247–5256. 37972589 10.1016/j.cub.2023.10.060

[ece370165-bib-0123] Trystram, C. , Rogers, K. M. , Soria, M. , & Jaquemet, S. (2017). Feeding patterns of two sympatric shark predators in coastal ecosystems of an oceanic Island. Canadian Journal of Fisheries and Aquatic Sciences, 74, 216–227. 10.1139/cjfas-2016-0105

[ece370165-bib-0124] UNEP‐WCMC , WorldFish Centre , WRI , & TNC . (2021). *Global distribution of coral reefs, compiled from multiple sources including the Millennium Coral Reef Mapping Project* (4.1) [Computer software]. 10.34892/t2wk-5t34

[ece370165-bib-0125] Villard, P. , Bretagnolle, V. , & Borsa, P. (2015). Segregation in diet between black Noddy (*Anous minutus*) and Brown Noddy (*A. stolidus*) from the southern lagoon of New Caledonia. Pacific Science, 69(2), 197–204. 10.2984/69.2.5

[ece370165-bib-0126] Walker, T. A. (1991). The distribution, abundance and dispersal by seabirds of Pisonia grandis. Atoll Research Bulletin, 350, 1–23.

[ece370165-bib-0127] Weimerskirch, H. , de Grissac, S. , Ravache, A. , Prudor, A. , Corbeau, A. , Congdon, B. , McDuie, F. , Bourgeois, K. , Dromzée, S. , Butscher, J. , Menkes, C. , Allain, V. , Vidal, E. , Jaeger, A. , & Borsa, P. (2020). At‐sea movements of wedge‐tailed shearwaters during and outside the breeding season from four colonies in New Caledonia. Marine Ecology Progress Series, 633, 225–238. 10.3354/meps13171

[ece370165-bib-0128] Weimerskirch, H. , Le Corre, M. , Jaquemet, S. , Potier, M. , & Marsac, F. (2004). Foraging strategy of a top predator in tropical waters: Great frigatebirds in the Mozambique Channel. Marine Ecology Progress Series, 275, 297–308. 10.3354/meps275297

[ece370165-bib-0129] Yarlett, R. T. , Perry, C. T. , Wilson, R. W. , & Philpot, K. E. (2018). Constraining species–size class variability in rates of parrotfish bioerosion on Maldivian coral reefs: Implications for regional‐scale bioerosion estimates. Marine Ecology Progress Series, 590, 155–169. 10.3354/meps12480

