# Supplementary material for: A framework and review of evidence of the importance of coral reefs for marine birds in tropical ecosystems
Source: Ecol Evol. 2024 Aug 21;14(8):e70165. doi: 10.1002/ece3.70165 (PMC11336204; doi:10.1002/ece3.70165)
Supplement: Supplementary file 3 — Appendix S3 [file ECE3-14-e70165-s001.docx]

**Supplementary Figures**

Supplementary Figure S1. Mean relative percent of reef fish items in the regurgitated samples of coastal and oceanic birds that live in the vicinity of coral reef habitat (< 25 km). Red line indicates 50% of prey items consumed originated in a coral reef. This figure shows what the results would demonstrate if reef fish larvae were treated separately as prey that are not reef-associated (based on the view that because this life stage is typically found off the reef, their consumption does not indicate bird predation adjacent to coral reef habitat).

**
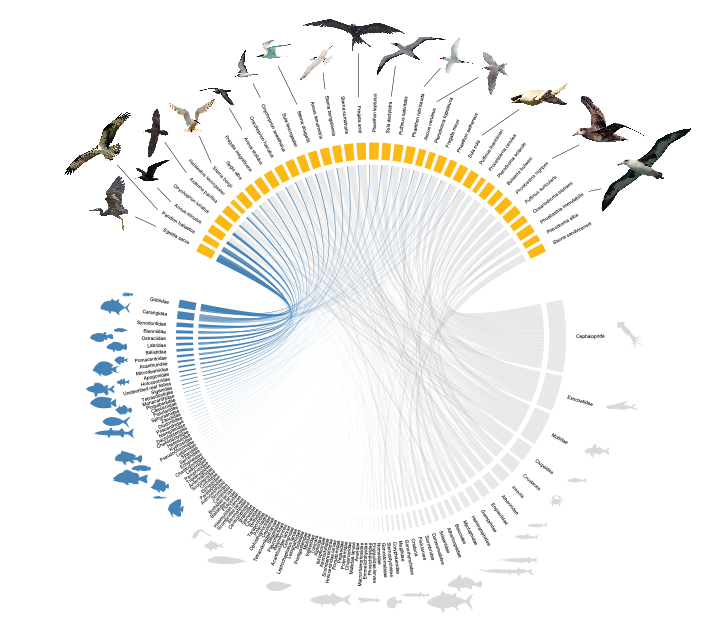
**

Supplementary Figure S2. Mean relative importance (%) of reef-derived prey items in the diet of 37 species of birds sampled on tropical islands within 25 km of coral reefs between 1964 and 2022. Yellow nodes represent birds, blue nodes represent families of reef-associated fishes, and grey nodes represent prey categories that are not reef-associated. Link thickness is proportional to the mean relative percent of prey items found in the regurgitated samples of birds. In this version of the figure, reef fish larvae are treated separately as prey that are not reef-associated to depict the alternative perspective that this life stage is typically found off the reef and therefore its consumption does not indicate bird predation in or adjacent to coral reef habitat. Bird images credits: Alan Schmierer, Lawrence Milovich, Forest & Kim Starr, Michael Morel, Mike Prince, Eric Dale, JJ Harrison, Paul Harrison, and Ariafrahman (please note that for review purposes a higher resolution version is provided as figure 4 in the supporting materials).

**Supplementary (on-line) Data**

**Appendix S1:** see linked Excel file

**Appendix S2:** see linked R script

**Appendix S3**: List of fish and bird species names recorded in the literature that were updated to match the current accepted taxonomic classification. Fish names were validated using the FishBase database and bird names were validated using the World Register of Marine Species and AviBase databases.

| **Name originally entered in the literature** | **Accepted name** | **Original data source/s** | **Database used for validation (r package)** |
| --- | --- | --- | --- |
| *Pranesus insularum* | *Atherinomorus insularum* | Harrison et al 1983 | FishBase (rFishbase::validate_names) |
| *Cypselurus atrisignis* | *Cheilopogon atrisignis* | Harrison et al 1983, Nascimento & Azevedo-Junior 2005 | FishBase (rFishbase::validate_names) |
| *Cypselurus spilonotopterus* | *Cheilopogon spilopterus* | Harrison et al 1983 | FishBase (rFishbase::validate_names) |
| *Cypselurus speculiger* | *Hirundichthys speculiger* | Harrison et al 1983, Seki & Harrison 1989, Nascimento & Azevedo-Junior 2005 | FishBase (rFishbase::validate_names) |
| *Cheilodactylus vittatus* | *Goniistius vittatus* | Harrison et al 1983, Seki & Harrison 1989 | FishBase (rFishbase::validate_names) |
| *Priacanthus cruentatus* | *Heteropriacanthus cruentatus* | Harrison et al 1983 | FishBase (rFishbase::validate_names) |
| *Hemipteronotus leclusei* | *Cymolutes lecluse* | Harrison et al 1983 | FishBase (rFishbase::validate_names) |
| *Remoropsis brachypterus* | *Remora brachyptera* | Harrison et al 1983 | FishBase (rFishbase::validate_names) |
| *Prognichthys gilberti* | *Hirundichthys rondeletii* | Harrison et al 1983 | FishBase (rFishbase::validate_names) |
| *Bleekeria gillii* | *Ammodytoides gilli* | Harrison et al 1983 | FishBase (rFishbase::validate_names) |
| *Stolephorus buccaneeri* | *Encrasicholina punctifer* | Harrison et al 1983 | FishBase (rFishbase::validate_names) |
| *Pteraclis velifer* | *Pteraclis velifera* | Harrison et al 1983 | FishBase (rFishbase::validate_names) |
| *Alutera scripta* | *Aluterus scriptus* | Harrison et al 1983 | FishBase (rFishbase::validate_names) |
| *Pegasus papilio* | *Eurypegasus papilio* | Harrison et al 1983 | FishBase (rFishbase::validate_names) |
| *Rhombochirus osteochir* | *Remora osteochir* | Harrison et al 1983 | FishBase (rFishbase::validate_names) |
| *Chromis vanderbilti* | *Pycnochromis vanderbilti* | Harrison et al 1983 | FishBase (rFishbase::validate_names) |
| Canthigasteridae (family) | Tetraodontidae (family) | Harrison et al 1983 | FishBase (web) |
| *Caranx hedlandensis* | *Carangoides hedlandensis* | Blaber et al 1995 | FishBase (rFishbase::validate_names) |
| *Lethrinus nematacanthus* | *Lethrinus genivittatus* | Blaber et al 1995 | FishBase (rFishbase::validate_names) |
| *Pristotis jerdoni* | *Pristotis obtusirostris* | Blaber et al 1995 | FishBase (rFishbase::validate_names) |
| *Cypselurus nigripennis* | *Hirundichthys speculiger* | Diamond 1975 (Tropicbirds) | FishBase (rFishbase::validate_names) |
| *Apogon cookii* | *Ostorhinchus cookii* | Jaquemet et al 2008 | FishBase (rFishbase::validate_names) |
| *Caranx crysos* | *Caranx crysops* | Nascimento & Azevedo-Junior 2005 | FishBase (rFishbase::validate_names) |
| *Haemulon aureolineatum* | *Haemulon aurolineatum* | Nascimento & Azevedo-Junior 2005 | FishBase (rFishbase::validate_names) |
| *Metavelifer multispinosus* | *Metavelifer multiradiatus* | Seki & Harrison 1989 | FishBase (rFishbase::validate_names) |
| *Parupeneus signatus* | *Parupeneus spilurus* | Surman & Wooller 2003 | FishBase (rFishbase::validate_names) |
| *Euleptorhamphus longirostris* | *Euleptorhamphus viridis* | Surman & Wooller 2003 | FishBase (rFishbase::validate_names) |
| *Sardinella lemura* | *Sardinella lemuru* | Surman & Wooller 2003 | WoRMS (worrms::wm_records_names) |
| *Velifer multiradiatus* | *Metavelifer multiradiatus* | Surman & Wooller 2003 | FishBase (rFishbase::validate_names) |
| *Puffinus newelli* | *Puffinus auricularis* | Ainley et al 2014 | WoRMS (worrms::wm_records_names) |
| *Sterna anaethetus* | *Onychoprion anaethetus* | Diamond 1983, Blaber et al 1995, Tayefeh et al 2014 | WoRMS (worrms::wm_records_names) |
| *Sterna fuscata* | *Onychoprion fuscatus* | Ashmole & Ashmole 1967, Brown 1975, Harrison et al 1983, Surman & Wooller 2003, Jaquemet et al 2008, Catry et al 2009 | WoRMS (worrms::wm_records_names) |
| *Thalasseus bengalensis* | *Sterna bengalensis* | Smith 1993 | WoRMS (worrms::wm_records_names) |
| *Thalasseus bergii* | *Sterna bergii* | Smith 1993 | WoRMS (worrms::wm_records_names) |
| *Ardenna pacifica* | *Puffinus pacificus* | Harrison et al 1983, Catry et al 2009 | WoRMS (worrms::wm_records_names) |
| *Anous cerulea* | *Anous ceruleus* | Harrison et al 1983 | AviBase (web) |
| *Phaeton lepturus* | *Phaethon lepturus* | Nascimento & Azevedo-Junior 2005 | WoRMS (worrms::wm_records_names) |
| *Haliaetus leucogaster* | *Haliaeetus leucogaster* | Smith 1985 | WoRMS (worrms::wm_records_names) |
